# NRPS gene dynamics in the wheat rhizoplane show increased proportion of viscosin NRPS genes of importance for root colonization during drought

**DOI:** 10.1128/msphere.00852-24

**Published:** 2025-09-15

**Authors:** Ying Guan, Edmond Berne, Rosanna Catherine Hennessy, Paolina Garbeva, Mette Haubjerg Nicolaisen, Frederik Bak

**Affiliations:** 1 Department of Plant and Environmental Science, University of Copenhagen, Frederiksberg C, Denmark; 2 Bioengineering Department, Polytech Nice Sophia, University Cote d’Azur, Nice, France; 3 Microbial Ecology, Netherlands Institute of Ecology (NIOO-KNAW), Wageningen, the Netherlands; University of Michigan, Ann Arbor, Michigan, USA

**Keywords:** CLPs, secondary metabolites, plant-microbe interactions, root microbiome, biosynthetic gene cluster, chemical ecology, *Pseudomonas *sp.

## Abstract

Secondary metabolites are bioactive compounds, diverse in structure, with versatile ecological functions, including key roles in mediating interactions between microorganisms and plants. Importantly, these compounds can promote the colonization of plant surfaces, such as roots, or modulate root exudates to enhance microbial recruitment and establishment. However, owing to the vast diversity of secondary metabolites, their importance in plant root colonization—particularly under stress conditions, such as drought—remains unclear. To determine the involvement of some of these secondary metabolites in root colonization, we used amplicon sequencing targeting the adenylation domain of the non-ribosomal peptide synthases (NRPSs) and the 16S rRNA gene from the rhizoplane of wheat grown in soil under normal and drought stress conditions. Results showed that drought transiently affected the bacterial community composition and the NRPS composition in the rhizoplane. We observed that drought selected for distinct groups of siderophores from different taxonomical groups, enriching for *Streptomyces* and depleting *Pseudomonas* siderophores. In addition, drought enriched *Pseudomonas*-derived NRPS genes encoding viscosin, a cyclic lipopeptide with biosurfactant properties, indicating that compounds linked to motility and colonization provide a competitive advantage during rhizoplane colonization under drought stress conditions. This observation was experimentally confirmed using the viscosin-producing *P. fluorescens* SBW25 and its viscosin-deficient mutant. A higher abundance of SBW25 colonized the roots under drought stress conditions compared to the viscosin-deficient mutant. In summary, our work demonstrates the potential for amplicon sequencing of NRPS genes, coupled with *in planta* experiments, to elucidate the importance of secondary metabolites in root colonization.

**IMPORTANCE:**

To harness beneficial plant–microbe interactions for improved plant resilience, we need to advance our understanding of key factors required for successful root colonization. Bacterial-produced secondary metabolites are important in plant–microbe interactions; thus, targeting these genes generates new knowledge that is essential for leveraging bacteria for sustainable agriculture. We used amplicon sequencing of the NRPS A domain on the rhizoplane of wheat exposed to drought stress to identify important secondary metabolites in plant–microbe interactions during drought. We show that the siderophores respond differently to drought stress depending on taxonomic affiliation and that the potential to synthesize viscosin increases root colonization. Importantly, this study demonstrates the potential of amplicon sequencing of NRPS genes to reveal specific secondary metabolites involved in root colonization, particularly in relation to drought stress, and highlights how the resolution provided by this approach can link specific compounds to a specific stress condition in a soil system.

## INTRODUCTION

Plants depend on microbes for beneficial functions, such as nutrient acquisition, disease suppression, and reduction of abiotic and biotic stresses (1, 2). While root exudates are important for recruiting and shaping the rhizosphere microbiome, only a subset of the rhizosphere microorganisms can colonize the root surface, i.e., the rhizoplane (3). Attachment to the rhizoplane is an important first step for close plant-bacteria interactions (4). For successful rhizoplane colonization, bacteria rely on a range of strategies (4), e.g., motility, production of antibiotics, and the production of a broad array of secondary metabolites.

One group of secondary metabolites, non-ribosomal peptides (NRPs), has been found to provide a variety of functions of importance for plant–microbe interactions, including antimicrobial activity (5), motility (6–8), biofilm formation (7, 9), root colonization (10, 11), and the induction of systemic resistance (ISR) in plants (12, 13). However, NRPs have been studied primarily in *Pseudomonas* and *Bacillus* (14), and their involvement in plant–microbe interactions under natural soil conditions remains largely unknown.

Given the roles of NRPs in shaping plant–microbe interactions, the composition of NRP synthases (NRPSs) and its temporal dynamics could provide cues to their specific mechanistic importance during root colonization. For example, Aleti et al. (15) showed a change in siderophore composition between emergence and senescence in potato, suggesting that different iron scavenging strategies are advantageous at distinct growth stages. Yet, this is the only study of temporal dynamics of NRPS genes in the root zone.

In addition, NRPs could provide an advantage for rhizoplane colonization during drought, due to their role in biofilm formation. At the same time, the production of NRPs, such as biosurfactants, can improve soil water distribution by reducing surface tension, thereby enhancing soil water retention and ultimately increasing plant drought tolerance (16–19). However, no studies have addressed the effect of drought stress on the composition of NRPs at the root surface. Such studies are needed to disentangle the impact of NRPs in root colonization during drought.

NRPs are synthesized by modular non-ribosomal peptide synthetases (NRPSs). Each module incorporates a specific amino acid into the peptide chain, starting with the adenylation (A) domain which selects and activates the amino acid. The specificity of the A domain is a key determinant for the resulting peptide’s sequence and function, and it can be targeted through amplicon sequencing. Recently, culture-independent approaches, and in particular, amplicon sequencing of NRPS gene clusters, have shed light on the composition of these genes in the rhizosphere of potato, populus, tomato, lettuce, cucumber, and wheat (15, 20–23); yet, there is little knowledge on the composition of NRPS genes on the rhizoplane.

Here, we used this novel culture-independent sequencing approach to study the rhizoplane—defined as the root surface and closely adhering soil (24, 25)—NRPS composition during the early growth stages of wheat under drought stress and subsequent recovery in natural soil systems. Winter wheat was grown under controlled conditions for six weeks, either under well-watered conditions or with a two-week drought regime introduced after two weeks of growth. Using amplicon sequencing of the 16S rRNA gene and the NRPS A domain, we specifically aimed to (1) characterize the temporal dynamics of NRPS diversity and composition in the rhizoplane of well-watered wheat plants and (2) assess the effect of drought on the NRPS composition in the rhizoplane, focusing particularly on the NRPS genes annotated to *Pseudomonas*. With the viscosin NRPS genes being the most abundant among the annotated *Pseudomonas* NRPSs, we complemented our findings with an experiment to further evaluate the importance of viscosin for root colonization under drought using *Pseudomonas fluorescens* SBW25 as a model strain.

## RESULTS

### Drought transiently impacts bacterial community taxonomic composition and diversity

We grew winter wheat (cv. Sheriff) for a duration of six weeks under controlled conditions in a growth chamber. The soil was collected from a nearby agricultural field and mixed with sterile sand (3:1; from here on “soil”). Drought was imposed on two-week-old wheat seedlings by withholding water for two weeks, after which water was added to alleviate the drought stress (Fig. S1). Control plants received continuous watering throughout the entire experiment.

**Fig 1:**
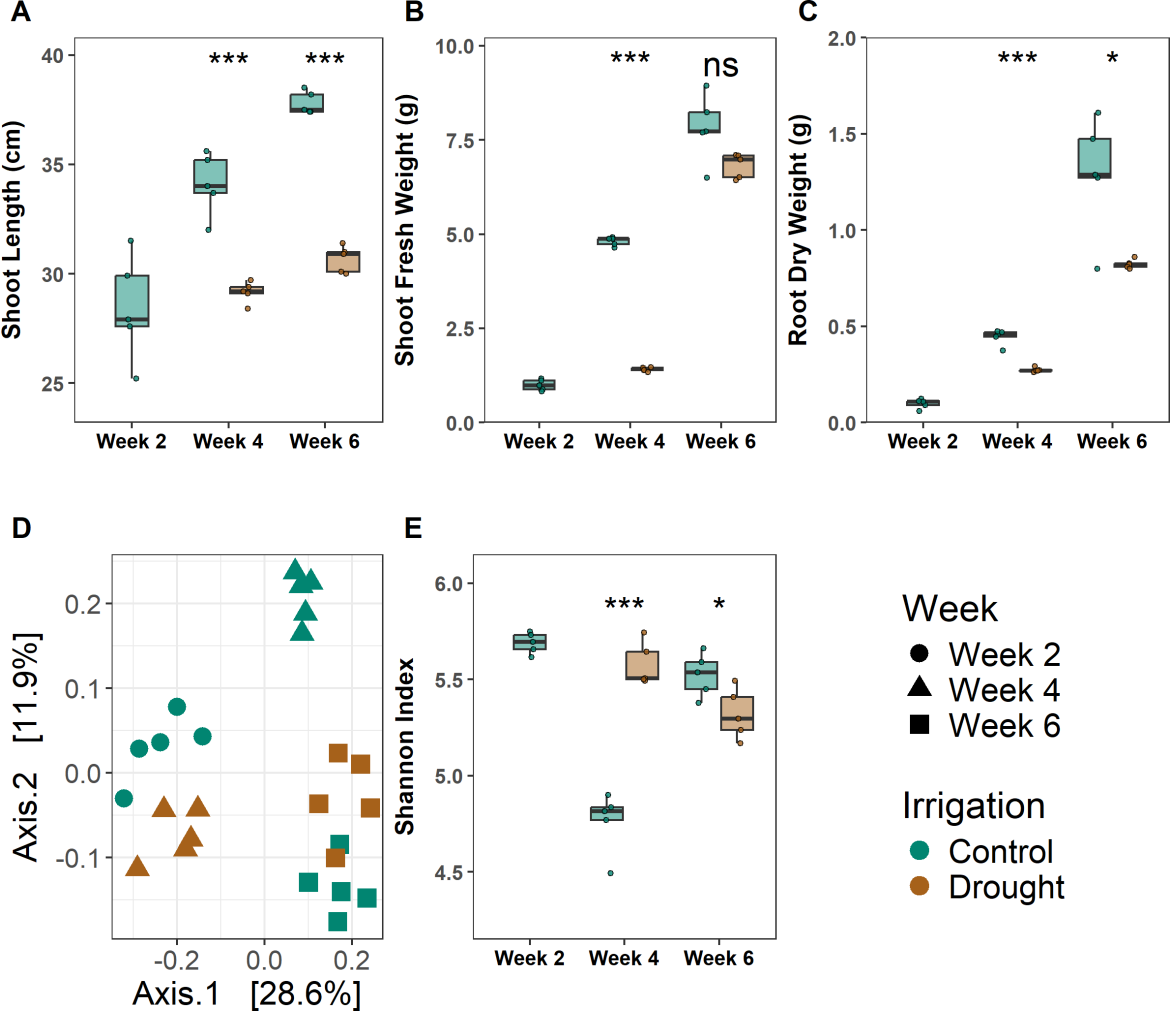
The effect of drought on plant growth before, during, and after drought. The shoot length (**A**), shoot fresh weight (**B**), and root dry weight (**C**) at different sampling times after sowing. Control: well-watered plants. Drought: drought stressed plants. (**D**) PCoA of Bray-Curtis dissimilarities of the bacterial community based on 16S rRNA amplicon sequencing. (**E**) Shannon diversity. Each box plot represents data from five replicates. Each dot represents a sample point. Asterisks in (**A–D**) indicate a statistically significant difference between the two groups (drought and control), which was derived from the *t*-test: *P < 0.05, **P < 0.01, and ***P < 0.001. The horizontal bars within boxes represent medians. The tops and bottoms of boxes represent the 75th and 25th percentiles, respectively. The upper and lower whiskers extend to data no more than 1.5 × the interquartile range from the upper edge and lower edge of the box, respectively.

Drought-stressed plants showed reduced shoot length, shoot fresh weight, and root dry weight at four weeks compared to the control plants (Fig. 1A through C). In addition, a reduction in chlorophyll content (*t*-test, *P* < 0.001), as well as higher peroxidase dismutase (POX) (*t*-test, *P* < 0.001), was observed (Fig. S2). No difference in superoxide dismutase (SOD) activities was recorded compared to control plants (Fig. S2). At six weeks, following two weeks of re-watering, drought-stressed plants remained growth impaired but showed signs of recovery in shoot fresh weight (Fig. 1A through C). Taken together, this shows that the treatment did impose a drought stress response during the experiment.

To determine the effect of the drought stress on bacterial rhizoplane communities, we initially performed 16S rRNA gene amplicon sequencing targeting the V5–V7 regions. We obtained 1,691 amplicon sequence variants (ASVs) with a median sequencing depth of 19,328 reads per sample, ranging from 12,905 to 28,215 reads per sample. Rarefaction curves indicated that sufficient sequencing depth was reached (Fig. S3). Drought changed the community composition (Fig. 1D; Table S1), but this change was transient as communities after six weeks, i.e., two weeks of re-watering, clustered together, as indicated by the positive interaction between time and water level (PERMANOVA, *R*^2^ = 0.11, *P* = 0.001, Table S1). Concomitantly, drought resulted in higher Shannon diversity in the rhizoplane community compared to control plants (*P* < 0.001, *t*-test) after four weeks, whereas Shannon diversity was higher in the control plants at week 6 (*P* = 0.043, *t*-test) (Fig. 1E).

At the ASV level, 48 ASVs increased in relative abundance during drought, while nine ASVs were more abundant in control plants (Corncob, FDR < 0.05, Fig. S4). Several bacterial genera, including *Bacillus*, *Streptomyces*, and *Kribbella*, increased in relative abundance on the rhizoplane of drought-stressed plants. In contrast, only seven genera, including *Massilia*, *Flavobacterium*, and *Arenimonas*, decreased in relative abundance during drought (Fig. S5). At week 6, only seven genera were differentially abundant between the two treatments, with *Streptomyces* still found at higher relative abundance in the plants exposed to drought (Fig. S6).

In conclusion, drought-induced plant effects were sustained throughout the experiment, while effects on the associated bacterial communities were transient, with bacterial communities for both treatments converging by week 6.

### Temporal dynamics is a key driver of NRPS composition and diversity

**Fig 2:**
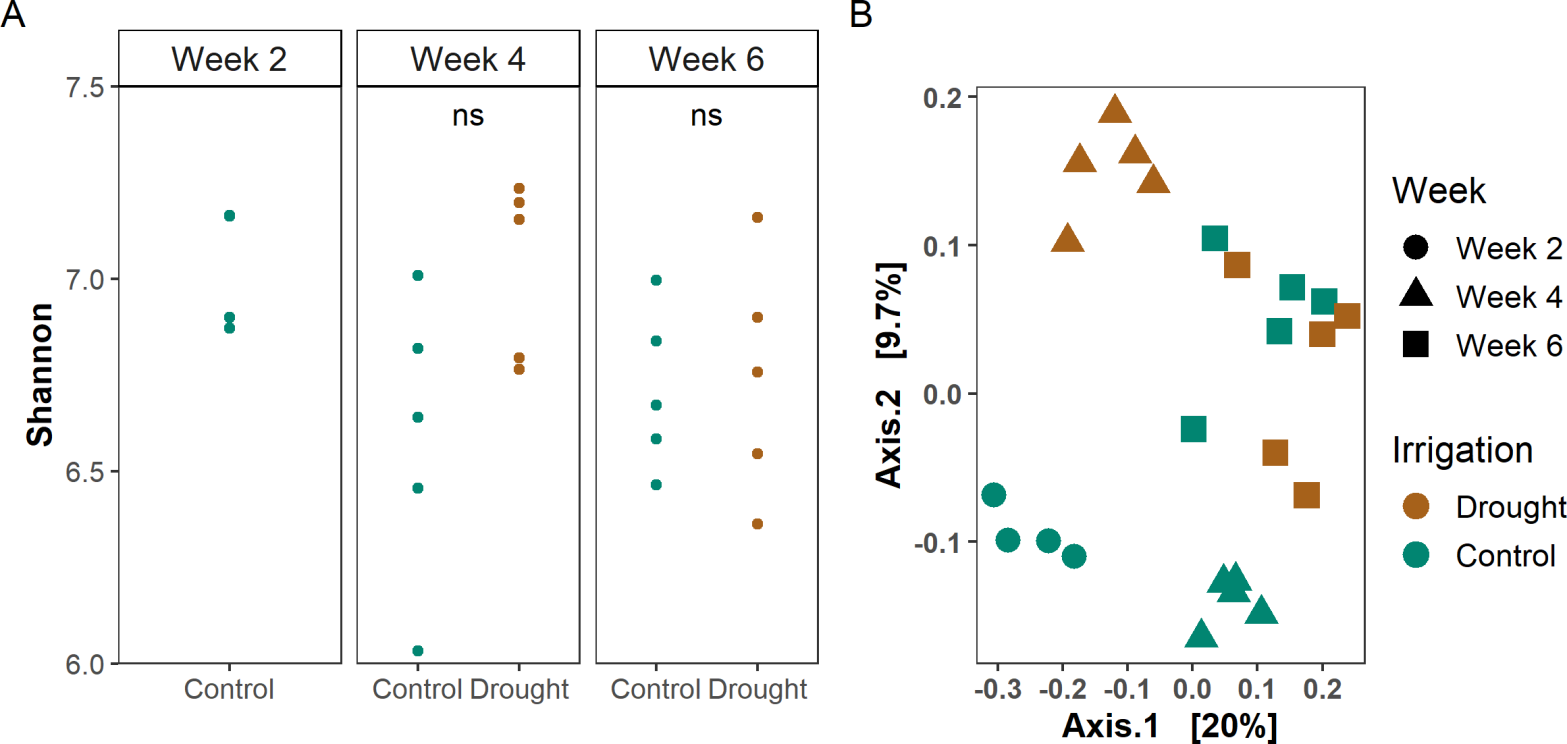
(**A**) Shannon index and (**B**) PCoA of rarefied NRPS ACs from the rhizoplane based on Bray-Curtis dissimilarities. NS, not significant (*P* > 0.05, *t*-test).

To go beyond taxonomic characterization of the microbiome, we performed amplicon sequencing targeting the NRPS A domain (23) from the rhizoplane at the three sampling points for both drought-stressed and non-drought (control) plants. After read processing, NRPS sequences were clustered into amplicon clusters (ACs) as in Tracanna et al. (23). The 25 samples contained 2,674,962 reads and 33,015 ACs. The median number of reads was 105,643 per sample, ranging from 22,354 to 230,381 reads. Rarefaction curves showed that the majority of the diversity was captured at around 40,000 reads per sample (Fig. S7). Neither the alpha diversity (Shannon index) (Fig. 2A) nor the richness (Fig. S8) of the NRPS domain A ACs differed between the sampled time points in the control plants. In contrast, developmental stage was a main driver of the NRPS AC composition (PERMANOVA, *R*^2^ = 0.23, *P* = 0.001, Fig. 2B; Table S2) in the control plants, explaining 23% of the variation.

### Drought alters NRPS composition to a lower extent than bacterial composition

As the two-week drought period changed the bacterial community in the rhizoplane (Fig. 1D), we investigated whether this observation was also applicable to the NRPS composition. Drought did not change the Shannon diversity (Fig. 2A) or the richness (Fig. S8) either at week 4 or week 6, contrasting the findings from the 16S rRNA amplicons. In line with observations at the taxonomic level, drought did, however, change the composition of the NRPS ACs (Fig. 2B) (PERMANOVA, *R*^2^ = 0.08, *P* = 0.005). Furthermore, the interaction between developmental stage and drought was also significant (PERMANOVA, *R*^2^ = 0.09, *P* = 0.001), indicating that the effect of drought on the NRPS AC composition was dependent on developmental stage. Based on visual inspection of the PCoA, the drought and control samples clustered together at week 6, suggesting that the effect of drought was transient (Fig. 2B). A comparison of the NRPS dissimilarity matrix with the 16S rRNA dissimilarity matrix showed an association between the two (Mantel’s *r* = 0.453, *P* = 0.001). However, only 45% of the variation in NRPS domain A diversity can be explained by the 16S rRNA taxonomic marker.

### Actinomycetota and Proteobacteria represent the largest proportion of NRPS ACs

Next, the NRPS reads were annotated using MiBig and Antismash databases in the dom2bgc pipeline (23), with the aim of assigning both taxonomy and function to the ACs. Despite using the most recent databases, only 15% and 16% of the ACs were annotated as NRPSs in the control and the drought-treated plants, respectively, across the three sampling times. Due to the high percentage of unassigned reads, we mapped the reads back to the model strains *P. fluorescens* SBW25. All reads mapping to the SBW25 genome aligned with known NRPS biosynthetic gene clusters, indicating high fidelity in the primers used.

**Fig 3:**
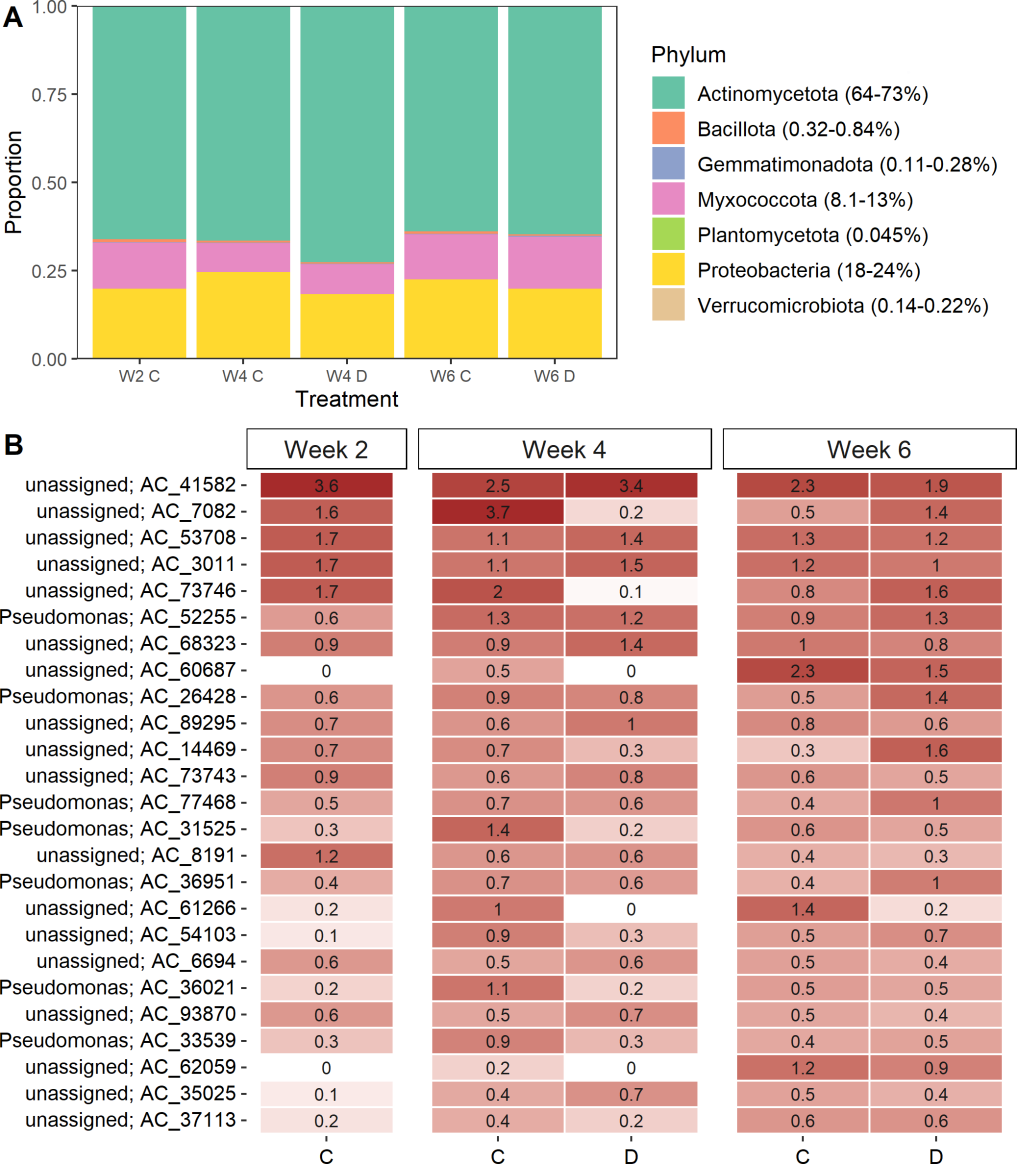
(**A**) Taxonomic assignment at the phylum level of the ACs in drought-stressed plants and control plants. Numbers in parentheses indicate the range of the relative abundance across samples. (**B**) Mean relative abundance of the 25 most abundant ACs across all sampling times and treatments in the rhizoplane (*n* = 5). C, control; D, drought.

Reads were taxonomically assigned to seven different phyla, with the majority (64-73%) of the ACs assigned to Actinomycetota. Proteobacteria and Myxococcota comprised 18–24% and 8–13%, respectively, depending on the watering regime (Fig. 3A). Together, Bacillota, Gemmatimonadota, and Verrucomicrobiota comprised ~1% of the annotated reads. For Bacillota, reads were assigned to *Paenibacillus*, *Thermoactinomyces*, *Tumebacillus*, and *Anoxybacillus*, whereas reads were only assigned to *Longimicrobium* and *Luteolibacter* for Gemmatimonadota and Verrucomicrobiota, respectively. NRPS ACs annotated to Planctomycetota (genus: *Aquisphaera*) were only detected in the drought-stressed plants at week 4 (<0.05% of annotated ACs).

Of the 25 most abundant NRPS ACs in the rhizoplane across developmental stage, seven were taxonomically assigned to *Pseudomonas* (Fig. 3B) and were functionally classified as NRPS. The rest were unassigned. Due to the low proportion of assigned amplicon clusters, we used BlastP to search the 25 most abundant ACs in the MiBig database v.3.1 (Table S3). The most abundant (amplicon_cluster_41582) was classified as Pf-5 pyoverdine. Of the *Pseudomonas*-associated NRPS reads, six of the seven were assigned to pyoverdine-related compounds (Table S3). Despite our effort, 10 of the 25 most abundant amplicon clusters did not represent known biosynthetic gene clusters, indicating a potentially high and untapped abundance of novel compounds in the rhizoplane.

To determine which ACs were enriched or depleted on a temporal scale, we tested differentially abundant ACs in the control plants using corncob (26). This showed that histicorrugatin, pyoverdines, and unassigned ACs from *Pseudomonas* increased from week 2 to week 4 (Fig. S9), coinciding with a subtle increase in relative abundance of *Pseudomonas* (Fig. S10). Pyoverdine, not assigned to any taxa, together with bacillibactin and occidiofungin, decreased between week 2 and week 4 (Fig. S9). In total, 108 ACs changed in relative abundance from week 2 to week 4. In contrast, only 39 ACs differed in relative abundance between week 4 and week 6, indicating a less dynamic functional microbiome at later developmental stages. Histicocorrugatin and pyoverdine from *Pseudomonas*, together with an unassigned phenalamide, decreased from week 4 to 6 (Fig. S11), while an omnipeptin assigned to *Saccharotix* increased in relative abundance. However, the majority of ACs that increased were either taxonomically or functionally unassigned.

### Drought reduces the relative abundance of *Pseudomonas* pyoverdine-related NRPS genes in the rhizoplane

**Fig 4:**
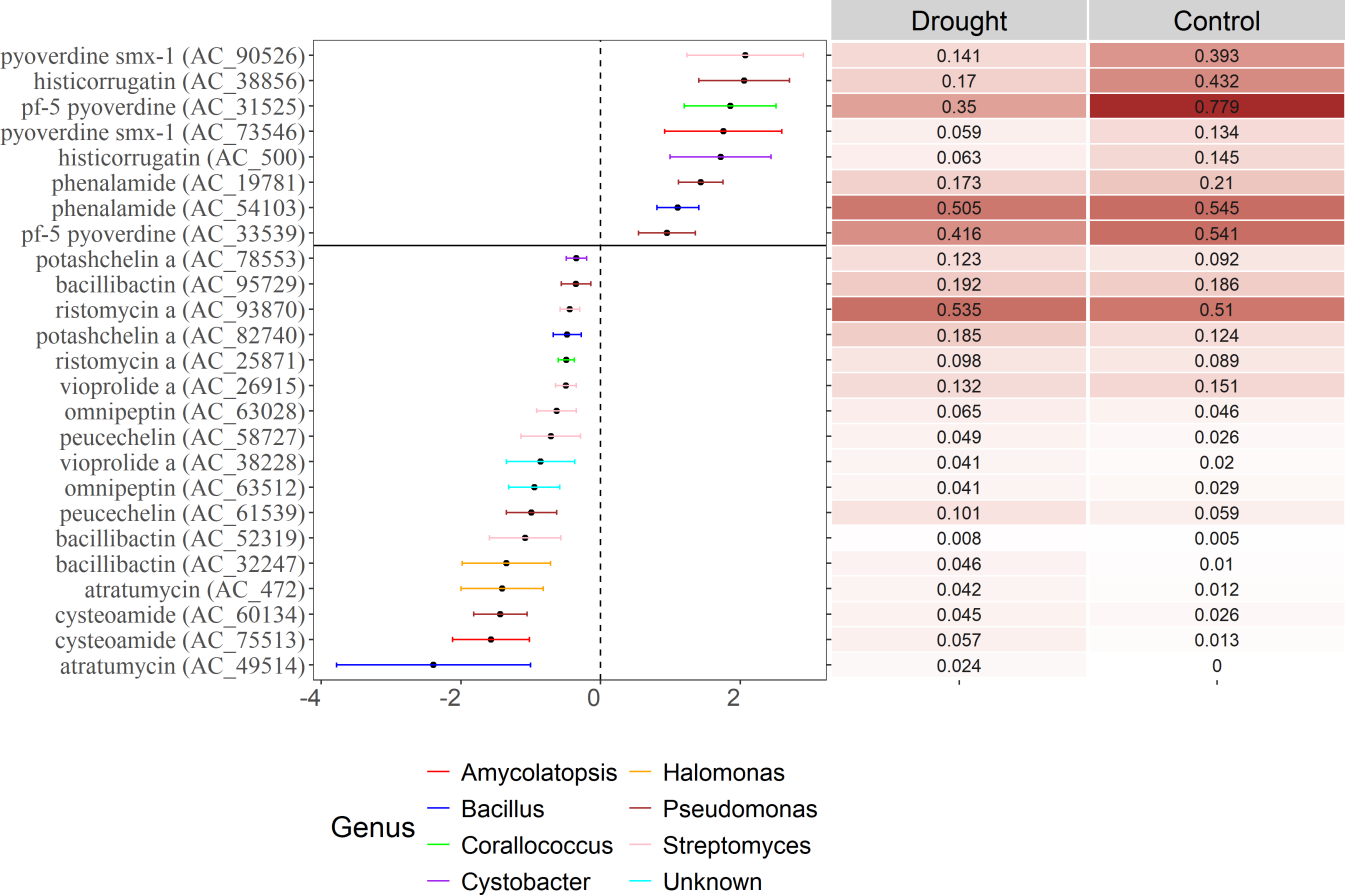
Differential abundance test (corncob, FDR < 0.05), right side shows the ACs depleted during drought, and left side shows the ACs enriched during drought. Mean relative abundances of the ACs in the rhizoplane at week 4 are shown in the heatmap on the right (*n* = 5). Only ACs that were annotated using MiBig and found at least twice are shown. See Fig. S12 for a full list of differential abundant ACs.

We then asked which ACs were enriched or depleted when plants were exposed to drought. In week 4, drought stress increased the relative abundance of 64 ACs and decreased the relative abundance of 37 ACs (Fig. S12). The majority of ACs were unassigned using the dom2bgc pipeline among the differentially abundant ACs at week 4. However, we identified eight ACs taxonomically assigned to *Streptomyces*, one assigned to *Rhodococcus*, one assigned to *Lentzea,* and two assigned to *Saccharotrix* among the ACs enriched in the drought-stressed plants at week 4. Contrastingly, we found seven *Pseudomonas*-assigned ACs, one *Mycolibacterium*-assigned AC, and two *Pseudoxanthomonas*-assigned ACs that were depleted during drought (Fig. S12). They were functionally annotated with a BlastP search against the MiBig database, and we identified genes encoding for bacillibactin, peuchelin, and potashchelin as ACs with highest prevalence among the differentially abundant ACs in the drought-stressed rhizoplane samples (Fig. 4; Table S4). These compounds are siderophores taxonomically assigned to *Bacillus*, *Streptomyces*, and *Halomonas*. Conversely, *Pseudomonas* genes coding for siderophores, such as pyoverdine, and histicocorrugatin decreased in relative abundance during drought (Fig. 4; Table S4). One week later, 11 ACs were enriched, and 11 ACs were depleted in the drought-stressed plants compared to the control plants (Fig. S13). Most of the ACs with a relatively higher abundance in the drought-stressed plants in week 6 were genes encoding pyoverdines.

To complement this analysis, we identified core ACs that were only present in drought-stressed or control plants (unique ACs). Here, we identified that the drought-stressed plants hosted a higher number of unique ACs (10) compared to the control plants (3) at the end of the drought period (week 4) (Table S5). Corroborating the results from the differential abundance test, coelichelin encoding NRPS genes were among the unique ACs in the drought-stressed plants at week 4. Coelichelin is a siderophore encoded by *Streptomyces*. Together, this shows that siderophores produced by *Streptomyces*, *Bacillus*, and *Halomonas* are enriched during drought, while siderophores produced by *Pseudomonas* are depleted. The dynamics of the siderophores partly reflect the dynamics at the taxonomic level, with an increase in relative abundance of *Streptomyces*, *Bacillus*, and *Halomonas*, while the relative abundance of *Pseudomonas* did not change during drought compared to the control (Fig. S10).

### The NRPS-derived CLP viscosin produced by *Pseudomonas* enhances root colonization under drought stress

NRPs previously reported to play key roles in root colonization include CLPs produced by *Bacillus* and *Pseudomonas* (10, 11, 27, 28). However, this has only been demonstrated during well-watered conditions. To determine whether CLPs provide an advantage in root colonization under drought stress, we mined the NRPS amplicons for CLPs produced by *Pseudomonas* (29).

**Fig 5:**
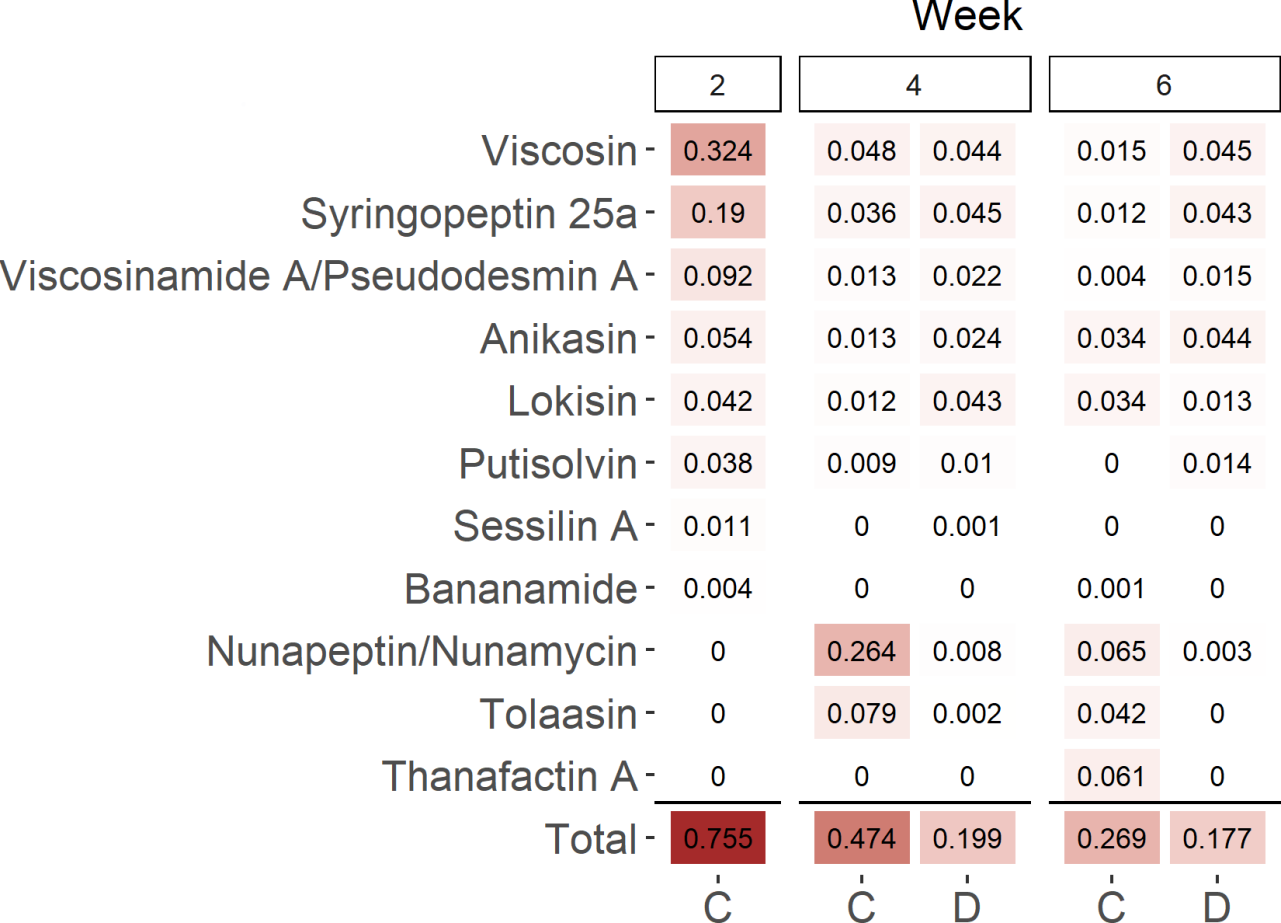
Proportion of reads assigned to different and the sum (Total) *Pseudomonas* CLPs out of all the reads in the NRPS amplicon data. Values are mean (*n* = 5). C, control; D, drought. Numbers above indicate week of sampling.

*Pseudomonas* comprised the highest proportion of the identified CLPs with a taxonomic assignment in the rhizoplane at all sampling times and water treatments (Fig. S14), despite the lower relative abundance compared to, for example, *Bacillus* (Fig. S10). The control plants harbored 2.5 times higher proportion of *Pseudomonas* CLP-related ACs than drought-stressed plants at week 4, while this difference was reduced to 0.5 times higher at week 6 (Fig. 5). This suggests that, in general, CLPs are not selected under drought stress. As we previously showed that the potential to synthesize viscosin provides an advantage in root colonization for *P. fluorescens* SBW25 (11), we were particularly interested in the NRPS ACs linked to this compound. Viscosin, together with viscosinamide, was one of the most relatively abundant *Pseudomonas* CLPs found across all samples in the rhizoplane (Fig. 5) and the most abundant at week 2. While the proportion of viscosin-coding ACs among all ACs was comparable between treatments at week 4, viscosin-coding ACs comprised 10% (0.048/0.474) of the *Pseudomonas* CLP-encoding ACs in the control plants and 22% (0.044/0.199) in the drought-stressed plants. This difference was even more noticeable after six weeks, where the viscosin-coding ACs comprised 25% (0.045/0.177) of the *Pseudomonas* CLP-encoding ACs in the drought-stressed plants compared to 6% (0.015/0.269) of these CLPs in control plants (Fig. 5). The increased proportion of viscosin-related ACs in drought-stressed plants indicates that *Pseudomonas* harboring the viscosin biosynthetic gene cluster colonizes the rhizoplane better than other CLP-producing pseudomonads. Thus, we hypothesized that the potential to produce viscosin would increase a strain’s root colonization potential during drought.

**Fig 6:**
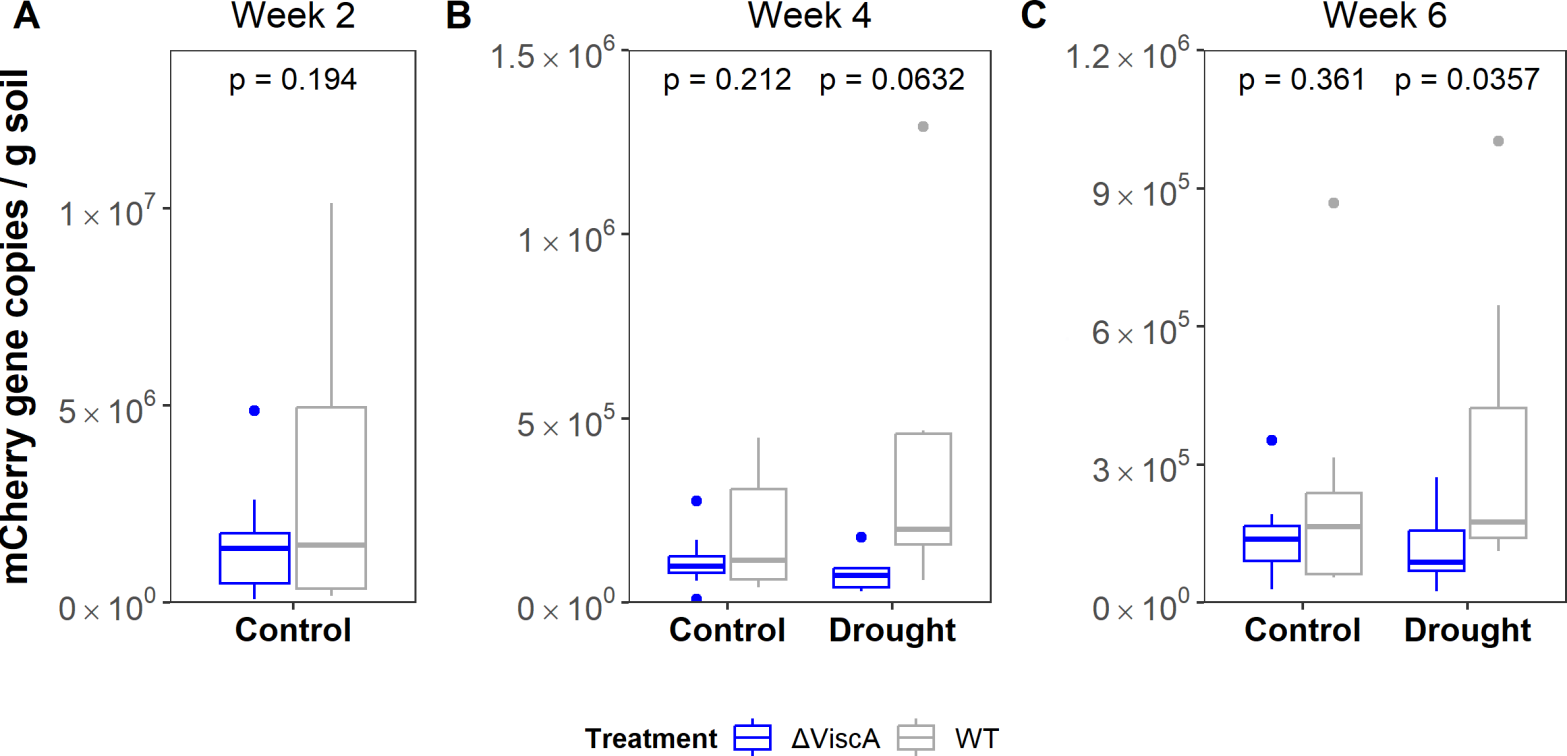
Absolute abundances of mCherry gene copies in the rhizoplane, at week 2 (**A**), week 4 (**B**), and week 6 (**C**). The box in the plot represents the interquartile range (IQR), with the center indicating the median and the box limits showing the upper and lower quartiles. The whiskers extend to 1.5 times the IQR from the quartiles. The *P* values indicate significance levels after *t*-test (*n* = 10).

To test this hypothesis, we inoculated surface-sterilized seedlings with a viscosin-producing strain, *P. fluorescens* SBW25 (WT), and a *viscA*-deficient mutant (Δ*viscA*) unable to synthesize viscosin and grew the plants as described above. We have previously shown that the WT colonizes wheat roots better under well-watered conditions (i.e., control plants) using both qPCR, CFU counts, and microscopy (11). Both WT and Δ*viscA* were chromosomally tagged with the mCherry gene, which was targeted with qPCR as a proxy for SBW25 abundance. The rhizoplane was sampled as described for the community analyses over a six-week period. A higher but non-significant abundance of mCherry genes per gram of rhizoplane soil was observed in the drought-stressed plants inoculated with the WT compared with plants inoculated with the Δ*viscA* mutant after four weeks (*t*-test, *P* = 0.06) (Fig. 6). At six weeks, this observation was even more pronounced with the WT showing higher colonization compared to the Δ*viscA* in drought-stressed plants (*t*-test, *P* = 0.03) (Fig. 6), despite a decrease in the number of mCherry genes from week 4 to week 6. At both sampling points, the mCherry abundances suggest similar rhizoplane colonization of the WT in the drought-stressed and the control plants. Whether inoculation with the WT or Δ*viscA* had an impact on plant health at week 4 was determined using chlorophyll, SOD, and POX activity measurements (Fig. S2). For control plants, no differences were observed among inoculations for chlorophyll or SOD and POX activities. In drought-stressed plants, inoculation with the WT increased the chlorophyll content compared to Δ*viscA*-inoculated (*P* = 0.04, *t*-test) and non-inoculated plants (*P* < 0.001, *t*-test), and the chlorophyll content was comparable with plants grown under controlled conditions. In addition, WT inoculation increased the SOD activity compared to non-inoculated plants (*P* = 0.037, *t*-test), but not compared to the Δ*viscA*-inoculated plants. Inoculation with WT or Δ*viscA* did not impact the POX activities. Hence, the inoculation with WT seemed to alleviate plant stress symptoms of low chlorophyll content. At the microbial side, this experiment supports the hypothesis that the ability to produce viscosin provides a competitive advantage in root colonization not only under normal conditions in natural soils, highlighting its general importance for plant root colonization by *Pseudomonas* species. Furthermore, it supports a role of viscosin during post-drought recovery. Additionally, we conclude that not all CLP results in an improved colonization during drought.

## DISCUSSION

Bacterial-produced NRPs play important roles in plant–microbe interactions (30). To date, research on NRPs in relation to plant growth has primarily focused on their growth-promoting activities or their biocontrol activity (29). However, their role in root colonization and specifically during drought stress has received limited attention.

Here, we exposed wheat (cv. Sheriff) to drought and determined the taxonomic and functional composition in the rhizoplane before, during, and after a drought period.

Overall, we observed that the NRPS composition in the rhizoplane changed over time. This has also been shown in the rhizosphere of potato (15) and reflects changes in overall community composition, as determined by 16S rRNA genes, as well as presumed changes in root exudates that attract bacteria. Nonetheless, the change in NRPS composition also indicates that the genes required for root compatibility change over time. Notably, NRPs, such as pyoverdines and histicorrugatin (*Pseudomonas* siderophores), changed with plant developmental stage, peaking at week 4. This suggests that the form or concentration of available iron around the roots changes with growth stage, although some of the increase may be attributed to an increase in *Pseudomonas*. This corroborates the findings by Aleti et al. (15), who also identified the relative abundances of siderophores to be dynamic at two different growth stages in potato.

A 2-week drought period changed the bacterial community, both at the taxonomic (16S rRNA gene) and the functional level (NRPS A domain). However, drought did not affect the alpha diversity and the richness of the NRPSs. Most of the NRPS ACs were unannotated, as also demonstrated in other studies applying NRPS or PKS amplicon sequencing in environments such as soil and rhizosphere (21, 23, 31). Due to the large proportion of unassigned ACs in the data set using the dom2bgc pipeline, we relaxed the cutoff criteria using BlastP against the Mibig database. Through this, we identified two NRPS-related gene groups that were affected by drought.

One group of NRPS genes affected by drought encoded iron-scavenging siderophores. A longer drought period increases the aerobic conditions in the soil, which in turn oxidizes the iron to form insoluble iron oxides (32), reducing availability for plants and microorganisms. Therefore, an effect on iron scavenging strategies during drought is to be expected. Future work could investigate whether, under drought conditions, the observed shifts in siderophore-encoding NRPS genes translate into measurable differences in siderophore production and iron acquisition.

*Pseudomonas-*encoded pyoverdines decreased during drought, whereas the relative abundance of *Pseudomonas* did not differ between treatments at four weeks. One potential explanation for this could be the production of yet unknown siderophores in rhizoplane-associated *Pseudomonas* strains, although further studies are warranted.

*Streptomyces*-encoded coelichelin, along with siderophores encoded by *Halomonas* and *Bacillus*, increased during the drought period. The latter coincided with an increase in relative abundance of *Streptomyces* and *Bacillus* in the 16S rRNA gene amplicons. These Gram-positive taxa are known for their ability to form spores, which protect them from desiccation during drought (33), and are enriched in the rhizosphere during drought for multiple plant species (34). While protection from desiccation offers a clear advantage during drought, the ability to scavenge iron may provide an additional advantage in the rhizoplane during drought, enabling access to an essential nutrient.

It has been shown that *Streptomyces* gain a competitive advantage under low iron availability conditions in soil through the secretion of different siderophores (35). This, together with the reduction of plant phytosiderophores during drought, has led Xu et al. (36) to propose that drought favors *Streptomyces* (or Actinobacteria in general) in the rhizosphere (36). In support of this, when plants were rewatered for two weeks, the relative abundance of several ACs coding for *Pseudomonas*-encoded pyoverdines increased. The affinity for iron varies between siderophores, with bacillibactin exhibiting a higher affinity than pyoverdine and most other siderophores, according to laboratory testing (37, 38). This provides Bacili with a theoretical advantage in taking up insoluble iron oxides under low availability, especially in drought conditions, giving them a competitive advantage over other bacteria.

While the work of Xu et al. (36) was performed using shotgun metagenomics, we demonstrate here the potential of using NRPS amplicons to infer the ecology in the root zone. Furthermore, this suggests that a relaxed cutoff criteria during BlastP was reasonable.

The second group of NRPS genes affected by drought was CLPs, a group of metabolites important in plant–microbe interactions (29). Species within *Pseudomonas* produce a vast collection of these compounds, as they have a role in biofilm dispersal, as antimicrobials, and in root colonization (29). Here, our analyses of the NRPS indicated that the ability to produce the CLP viscosin is an important trait for root colonization of wheat by *Pseudomonas* during drought. This was supported by the higher abundance of *P. fluorescens* SBW25 in the rhizoplane during and after exposure to drought compared to its viscosin-deficient mutant. This extends our previous findings of the role of viscosin in root colonization as it enhances root colonization at different watering regimes. In this work, we determined the abundance using qPCR targeting the mCherry gene, as this method was supported by CFU counts and microscopy images in a comparable setup (11). While research on the importance of CLP production in rhizoplane colonization in cereals is scarce, orfamide (another CLP) has also been shown to increase rhizoplane colonization in wheat (39). Viscosin likely functions as a wetting agent capable of enhancing solubility of nutrients and hydrophobic substrates, as proposed for other CLPs (40). For example, surface-active *P. fluorescens* and *P. putida* strains can increase leaf wetness and may enhance leaf attachment (41). Furthermore, syringafactins from the plant pathogen *P. syringae* can attract water vapor, thereby alleviating water stress on dry leaves (42). Thus, we speculate that *P. fluorescens* SBW25 uses viscosin as a wetting agent to enhance root attachment and increase nutrient availability. Whether this nutrient availability benefits the plant needs to be determined; however, inoculation with SBW25 increased chlorophyll content and the SOD activity of the leaves after four weeks of drought stress. An increase in SOD after inoculation with PGBR during drought stress has been observed before in potato (43). However, we cannot conclude whether it is the ability to synthesize viscosin or other beneficial effects of the SBW25 that led to increased chlorophyll content and SOD activity.

On a broader level, the composition of 16S rRNA gene and NRPS amplicons converged two weeks after the drought period ended. Rewetting after drought increases microbial biomass in the rhizosphere (44), likely due to accumulated dissolved organic carbon during the drought (45). Further, Karlowsky et al. (44) found that both Gram-positive and Gram-negative bacteria took up ^13^C-labeled carbon to the same extent after rewetting. These studies support our findings that rhizosphere bacteria respond evenly in carbon uptake to rewetting. However, at a finer resolution, we still saw a “legacy” effect of drought on the viscosin-encoding ACs, which were relatively more abundant two weeks after drought ended, as well as on other pseudomonad CLPs. This points to an underlying functional microdiversity, e.g., the ability to produce viscosin, within the microbial community supporting different ecotypes of the same species (46, 47). This is supported by the fact that only 45% of the taxonomic diversity explains the NRPS diversity observed. In summary, the effects of drought on the community level were transient, but the NRPS amplicons revealed that functionality was affected for a longer duration. Hence, new knowledge on the microdiversity beyond the taxonomic level is needed to gain a complete understanding of microbial interactions in the plant–soil interface.

Our data analysis identified numerous unassigned ACs, highlighting the need for further research in this area to strengthen currently available databases. Interestingly, NRPSs assigned to Verrucomicrobiota, Gemmatimonadetes, and Planctomyceota were recently linked to the synthesis of secondary metabolites in soil (48, 49), suggesting that they contribute an important diversity of secondary metabolites in the rhizosphere. Despite this inherent limitation, our study demonstrates the potential of amplicon sequencing of NRPS genes to reveal specific secondary metabolites involved in root colonization and in relation to drought stress, which can be further supported by experimental work.

Overall, our study shows that the root microbiome harbors a wide range of NRPS genes changing in composition during plant growth and under drought stress. Specifically, the differences in siderophore-encoding genes highlight the importance of identifying ecosystem-associated keystone metabolites. Furthermore, we demonstrated how an NRPS amplicon approach can be used to identify key metabolites, e.g., viscosin, in *Pseudomonas* required for root colonization during drought and further confirmed this finding by experimental work. Expanding our current repositories of NRPS clusters will enhance the use of these amplicons to resolve important ecological questions on the role of NRPs in shaping root colonization patterns and mediating plant–microbe interactions in the rhizosphere.

## MATERIALS AND METHODS

### Plant materials and growth conditions

The winter wheat cultivar Sheriff (Sejet Plant Breeding, Horsens, Denmark) seeds were used in this study. Initially, the seeds were submerged in sterile MilliQ water for an hour, then transferred to Petri dishes with dual layers of sterile filter paper, each saturated with 5 mL of sterile MilliQ water for germination. For optimal germination, the seeds were kept in the dark at room temperature for three days. Following this, the three-day-old germinated seedlings were root-dipped in sterile saline solution (0.9% NaCl) and transplanted into polyvinyl chloride (PVC) pots (24 cm in height and 7 cm in diameter) with drainage holes, each pot accommodating one plant. The pots were filled with approximately 1,000 g of non-sterile field soil premixed with sterile sand (DANSAND, Brædstrup, Denmark) at a 3:2 ratio (“soil” from here on). The field soil was gathered from the plow layer (0 to 25 cm) at the University of Copenhagen’s experimental farm in Taastrup, Denmark (55° 40′N, 12° 17′E). The soil was a sandy loam (170 g clay kg^−1^, 174 g silt kg^−1^, 362 g fine sand kg^−1^, 255 g coarse sand kg^−1^, and 17 g soil organic matter kg^−1^) (50).

The water content of the soil was maintained at 17% (wt/wt). Following the transplantation of the germinated seeds into the pots, the soil was supplemented with a nutrient solution containing Nitrogen–Phosphorous–Potassium (NPK 3-1-4) (Drivhusgødning, Park). Each pot received 0.83 mL of the nutrient solution, which contained 2.04% nitrate nitrogen (N), 0.3% ammonium nitrogen (N), 0.46% amide nitrogen (N), 0.69% phosphorus (P), 4.38% potassium (K), 0.08% sulfur (S), 0.06% magnesium (Mg), 0.033% iron (Fe), 0.013% manganese (Mn), 0.002% copper (Cu), 0.002% zinc (Zn), 0.0006% molybdenum (Mo), and 0.004% boron (B). Subsequently, the pots were positioned in the growth chamber that was set at 600 µmol m^−2^ s^−1^ light intensity, 60% humidity, a 16/8  h light/dark photoperiod, and a temperature of 19°C during the day and 15°C at night.

### Plant experiments under drought and well-watered conditions

Twenty-five plants were randomly divided into two distinct watering regimes: 10 plants for drought stress treatment and 15 for the well-watered control (“control” from here on). Sample collection took place at 14, 28, and 42 days post-sowing, covering a pre-drought, a drought, and a post-drought time interval. The experimental timeline is presented in Fig. S1. This experimental setup resulted in five biological replicates for each treatment and sampling time point. All plants were cultivated under well-watered conditions for 14 days, after which the drought group underwent a drought treatment for an additional 14 days by withholding water. During non-drought periods and for well-watered plants, distilled water was administered every other day to maintain a soil moisture content of 17% throughout the experiment. For plants subjected to drought stress, water was reintroduced to the PVC pots at the end of the drought period (28 days post-sowing) to facilitate plant recovery.

### Sampling of rhizoplane-associated microbial communities

Root sample collection and compartment processing were performed as described by (11) to obtain rhizoplane-associated microorganisms (the root surface and adhering soil) (24). Roots were washed in 25 mL sterile MilliQ water by shaking for one minute. Subsequently, roots were transferred to a new tube with 25 mL sterile MilliQ water and sonicated for one minute. After removal of the roots, the second wash was defined as the rhizoplane sample. Samples for DNA extraction were immediately flash-frozen in liquid nitrogen and kept on dry ice until storing at −80°C. Samples were freeze-dried (CoolSafe 100-9 Pro freeze dryer, LaboGene, Lynge, Denmark). All freeze-dried samples were stored at −20°C until DNA extraction. Root and shoot lengths were recorded at sampling. Subsequently, roots and shoots were dried at 60°C for three days before determining the dry weight.

### DNA extraction

Genomic DNA was extracted from 500 mg of rhizoplane samples using the FastPrep-24TM 5G bead-beating system (MP Biomedicals, Irvine, CA, USA), set at 6.0 m/s for 40 seconds and the FastDNA SPIN Kit for soil (MP Biomedicals), strictly following the manufacturer’s instructions. The DNA extracted from all samples was eluted in 60 µL elution buffer. The DNA’s purity and concentration were evaluated using a NanoDrop ND-1000 spectrophotometer (Thermo Fisher Scientific, Carlsbad, CA, USA). All DNA samples were stored at −20°C for subsequent quantitative PCR analysis and amplicon library preparation.

### Chlorophyll quantification

The chlorophyll content of leaves from control and drought-stressed plants was determined at 28 days post-sowing using a modified version of Liang’s method (51). The chlorophyll content was then calculated using Arnon’s classic equations (52) with absorbance values measured at 663 nm and 645 nm (full description in Supplemental material).

### Determination of superoxide dismutase (SOD) and peroxidase (POX) activities in wheat leaves

SOD and POX activities were measured at 28 days post-sowing, following a slightly modified version of the method previously described (53). SOD activity was measured by recording the reduction in the optical density at 560 nm of the nitro-blue tetrazolium (NBT) dye catalyzed by the enzyme. POX activity was assessed based on the increase in optical density at 470 nm resulting from the formation of tetra-guaiacol. We refer to the Supplemental material for full details.

### Strains and growth conditions

The viscosin-producing model strain *P. fluorescens* SBW25, along with its mutant strain that is deficient in viscosin production, was labeled using mCherry in a previous study (11). These strains were recovered from frozen glycerol stocks and cultured on Luria Broth Agar (LBA) plates, which consist of 1% tryptone, 0.5% yeast extract, 1% NaCl, and 1.5% agar, all supplemented with 10 µg mL^−1^ gentamicin. The plates were then incubated at 28°C for 48 hours. Subsequently, single colonies were transferred into 20 mL liquid LB culture tubes with 10 µg mL^−1^ gentamicin supplementation and incubated at 28°C with continuous shaking at 180 rpm.

### Preparation of bacterial suspension and inoculation on wheat seedlings

The bacterial cells from the overnight culture were obtained through centrifugation at 6,000 × *g* for five minutes to yield cell pellets. These pellets were subsequently washed twice and resuspended in 0.9% sterile NaCl. OD_600_ for each inoculum was standardized to 1.0, equivalent to approximately 5 × 10^8^ CFU mL^−1^. The roots of three-day-old germinated seedlings were immersed in either a *P. fluorescens* SBW25 mCherry suspension (WT) or a *P. fluorescens* SBW25Δ*viscA* mCherry suspension (Δ*viscA*) for two hours. Subsequent to the inoculation, all plants were cultivated as detailed above (*n* = 10). Briefly, sampling and DNA extraction were performed as described above.

### Quantitative PCR analysis

The quantification of WT and Δ*viscA* was executed using qPCR as described in (11). The specific primers utilized are listed in Table 1. The absolute abundance of the target *mCherry* gene was subsequently calculated based on a standard curve derived from a ten-fold serial dilution of DNA from SBW25::Tn7::mCherry cells. This standard curve exhibited a dynamic range spanning a ten-fold dilution series from 10^2^ to 10^8^ copies μL^−1^, with three technical replicates for each dilution. The efficiency ranged from 99.2% to 99.8%, and *R*^2^ values were >0.99 for all standard curves. In addition, the qPCR setup included three technical replicates of a non-template (nuclease-free water) negative control.

**TABLE 1:** Primers used in the study

Primers	Sequence	Reference
NRPS A3	5′-GCSTACSYSATSTACACSTCSGG-3′	(54)
NRPS A7R	5′-SASGTCVCCSGTSCGGTAS-3′	(54)
799F	5′-AACMGGATTAGATACCCKG-3′	(55)
1193R	5′-ACGTCATCCCCACCTTCC-3′	(56)
*mCherry*-Fw	5′-GCCCCGTAATGCAGAAGAAG-3′	(11)
*mCherry*-Rv	5′-GTGTAGTCCTCGTTGTGGGA-3′	(11)

### NRPS A domain and 16S rRNA amplicon sequencing

Primers A3F and A7R, detailed in Table 1, are used in a PCR reaction to amplify the NRPS A domains (54). A two-step PCR and a dual-indexing approach were employed for Illumina MiSeq sequencing. The PCR amplicons were produced using 15 µL of Platinum II Hot-start PCR Master Mix (Thermo Fisher Scientific), 0.6 µL of forward and reverse primers each (10 µM), 8.8 µL of nuclease-free water, and 5 µL of template. The PCR thermocycler protocol encompassed an initial denaturation phase at 95°C for two minutes, followed by 33 cycles of 95°C for 15 seconds, 55°C for 15 seconds, and 72°C for 15 seconds, with a final elongation step at 72°C for five minutes. The PCR amplification was verified via 1.5% agarose gels with loading buffer, and the PCR products were subsequently purified using AMPure XP beads (Beckman Coulter Inc. Brea, CA, USA). The purified DNA concentration was reassessed with a NanoDrop ND-1000 spectrophotometer (Thermo Fisher Scientific, Carlsbad, CA, USA) and a Qubit 2.0 fluorometer using the High-Sensitivity DNA assay (Thermo Fisher Scientific). Finally, the subsequent library construction and Illumina MiSeq sequencing (2 × 300 bp) were undertaken by the Erasmus Center for Biomics (Rotterdam, Netherlands).

The V5-V7 region of the bacterial 16S rRNA gene was amplified using primers 799F (55) and 1193R (56) (Table 1). Notably, this primer pair results in minimal amplification of plant mitochondria and chloroplast DNA (57). The ZymoBIOMICS Microbial Community DNA Standard (Zymo Research, Irvine, CA, USA), pure culture SBW25 DNA, and water negative controls were included. A two-step dual-indexing approach was employed for Illumina MiSeq sequencing, as described in (11). The subsequent library construction and Illumina MiSeq sequencing (2 × 300 bp) were performed by Eurofins Genomics (Ebersberg, Germany).

### Sequence processing

The raw amplicon sequence reads were processed using the DADA2 pipeline (version 1.30) (58). Aside from a few parameter alterations, all other settings were retained as default. Specifically, for 16S rRNA gene reads, based on sequence quality, filtering was applied using default parameters, except for ‘trimLeft’ and ‘truncLen’. Primers were removed using ‘trimLeft’ = 19/18, depending on primer length, and reads were truncated using ‘truncLen’ = 280/200 for the forward and the reverse reads, respectively, to avoid poor quality and ambiguous sequences. Chimeras were removed after merging denoised pair-end sequences. Each unique 16S rRNA amplicon sequence variant (ASV) was classified according to the SILVA database (version 138.1) (59). Non-bacterial ASVs, including chloroplasts and mitochondrial reads, were filtered out, and the mock community and negative controls were checked for contamination. For NRPS A domain reads, solely the forward reads were employed, as recommended in (23). Reads were truncated at 250 nt and quality filtered using maxEE = 7. NRPS A domain reads were annotated using a modified version of the dom2bgc pipeline (23). In brief, forward NRPS reads were translated into six frames and aligned the reads. Translated protein sequences were clustered based on Euclidean distances (23) and annotated using the most recent versions of MiBig (v. 3.1) (60) and antiSmash databases v.7.0.0 (61). In addition to the annotation performed using the dom2bgc pipeline, we performed a BlastP search of the NRPS ACs against the most recent MiBig database v.3.1 (60), using an E value threshold of 10^–20^.

### Data analysis and statistics

Statistical analysis was performed in R version 4.3 (62). Differences in plant parameters and gene copy numbers between the drought-stressed and control plants were analyzed by an unpaired *t*-test (*P* < 0.05). *P* values were adjusted for multiple comparisons using “BH.” ANOVA was used to test for differences among different inoculations (*P* < 0.05).

For microbiome diversity and composition analyses, we used the R packages phyloseq version 1.46 (63) and ampvis2 version 2.8.9 (64). The amp_rarecurve function in the ampvis2 package was used to generate rarefaction curves for each sample. For the determination of alpha diversity, richness, beta diversity, and unique NRPS ACs, data were rarefied to an even depth (42,000 reads based on rarefaction curves) using the mean values of 100 iterations. The alpha diversity and richness for the 16S rRNA and NRPS amplicons were determined using the Shannon diversity and CHAO1 indices with phyloseq_estimate_richness. Significance testing of the Shannon diversity was done with a *t*-test (*P* < 0.05). For the NRPS A domain amplicons, the sample I23.1089.E04 was excluded from the analysis due to low read number (22,354 reads).

A principal coordinates analysis (PCoA) was performed using ordinate() in phyloseq (based on vegdist() from vegan) with rarefied data and a Bray-Curtis dissimilarity matrix. Permutational Multivariate Analysis of Variance (PERMANOVA), based on Bray-Curtis dissimilarity matrix, was used to test the effects of different factors (sampling time and watering treatment) and their interactions on the beta diversity of communities in vegan version 2.6.6.1 (65). Dissimilarity matrices of 16S rRNA amplicons and NRPS amplicons were compared using a Mantel test (Pearson correlations) in vegan. The differential abundance of ASVs between groups was determined using beta-binomial regression with the corncob package, version 0.4.1 (26). Only ASVs or ACs with an estimated differential abundance of < −1 or >1, and *P*-values adjusted for multiple testing <0.05 (FDR < 0.05), were considered significant. We searched for ACs unique to either the drought-stressed or control plants among the core ACs in the rhizoplane. Here, we defined core ACs to be present in at least four out of five replicates. The core ACs of one treatment were then compared to all ACs from the other to determine unique core ACs. Identification of NRPS known to be produced by *Pseudomonas* was performed using the complete list of compounds presented in (29).

## Data Availability

All raw sequencing data used in this study had been deposited in the NCBI Sequence Read Archive (SRA) database under accession codes PRJNA987606 (16S rRNA) and PRJNA987694 (AD). The scripts describing data treatment are available at https://github.com/Edmondbrn/Linking_microbial_community_structure_with_function.

## SUPPLEMENTAL MATERIAL

Supplemental material msphere.00852-24-s0001.docx Fig. S1 to S14 and supplemental methods.

Supplemental tables msphere.00852-24-s0002.xlsx Tables S1 to S5.

ASM does not own the copyrights to Supplemental Material that may be linked to, or accessed through, an article. The authors have granted ASM a non-exclusive, world-wide license to publish the Supplemental Material files. Please contact the corresponding author directly for reuse.
