# Supplementary material for: NRPS gene dynamics in the wheat rhizoplane show increased proportion of viscosin NRPS genes of importance for root colonization during drought
Source: mSphere. 2025 Sep 15;10(10):e00852-24. doi: 10.1128/msphere.00852-24 (PMC12570501; doi:10.1128/msphere.00852-24)
Supplement: Supplemental material — Fig. S1 to S14 and supplemental methods. [file msphere.00852-24-s0001.docx]

**Supporting information for:**

NRPS gene dynamics in the wheat rhizoplane show increased proportion of viscosin NRPS genes of importance for root colonization during drought

Ying Guan^1a^, Edmond Berne^2^, Rosanna Catherine Hennessy^1^, Paolina Garbeva^3^, Mette Haubjerg Nicolaisen^1*#^, Frederik Bak^1#^

^1^ Department of Plant and Environmental Science, University of Copenhagen, Frederiksberg C, Denmark

^2^ Bioengineering Department, Polytech Nice Sophia, University Cote d’Azur, Nice, France

^3^ Microbial Ecology, Netherlands Institute of Ecology (NIOO-KNAW), Wageningen, Netherlands

^a^ Present address: College of Resources and Environmental Science, Nanjing Agricultural University, Nanjing, China

* Correspondence: [meni@plen.ku.dk](mailto:meni@plen.ku.dk)

# These two authors contributed equally


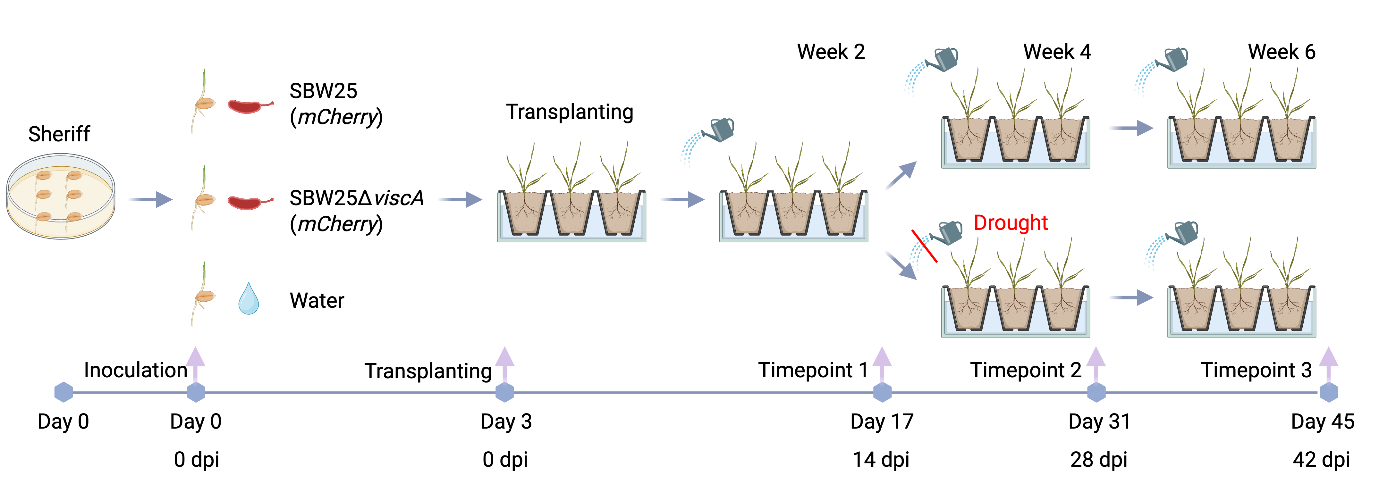


Figure S1. The timeline for plant experiments under drought and well- water conditions. Timeline is shown below, DPI (Days Post Inoculation).


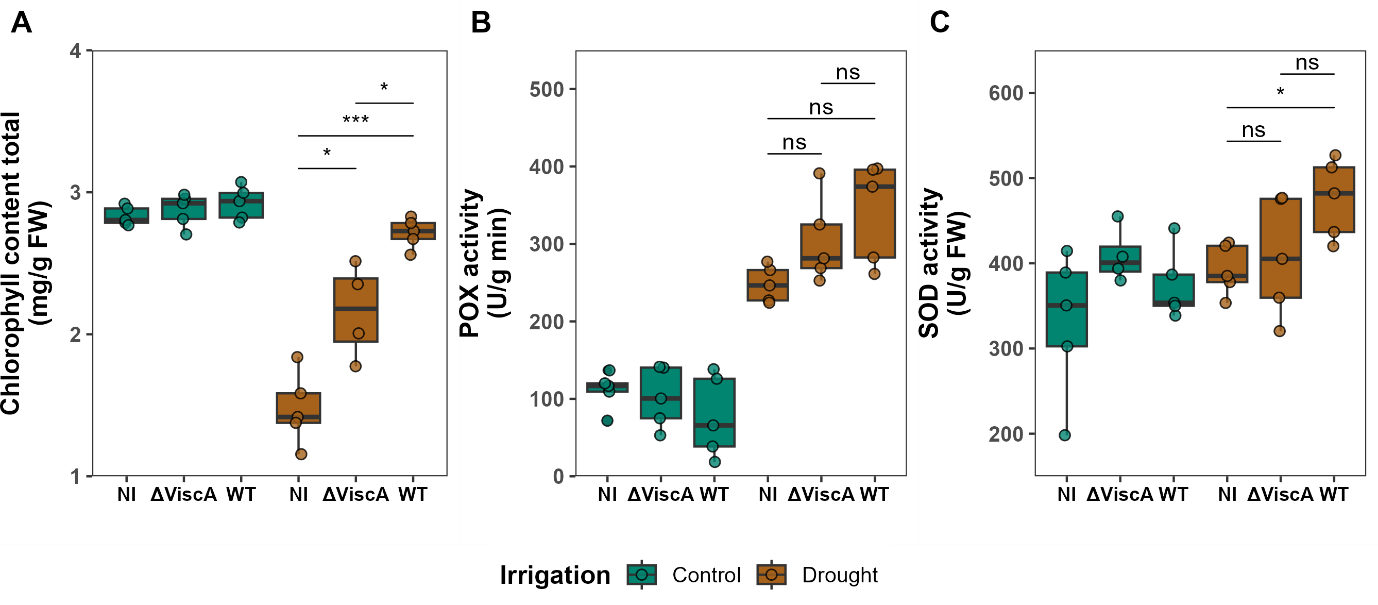
Figure S2. The effects of inoculation with SBW25 and mutant on chlorophyll content, POX and SOD activity were assessed 4 weeks post sowing drought stress and well-watered conditions. **A)** total chlorophyll content. **B)** POX activity. **C)** SOD activity. Each box plot represents data from five replicates. Each dot represents a sample point. The horizontal bars within boxes represent medians. The tops and bottoms of boxes represent the 75th and 25th percentiles, respectively. The upper and lower whiskers extend to data no more than 1.5× the interquartile range from the upper edge and lower edge of the box, respectively. NI: Non-inoculated control, ΔviscA: ΔviscA Mutant, WT: SBW25. ANOVA was used to determine whether inoculation was significant (p < 0.05) within irrigation. Asterisks indicate a statistically significant difference between the two inoculations (t-test) *p < 0.05 and **p < 0.01.


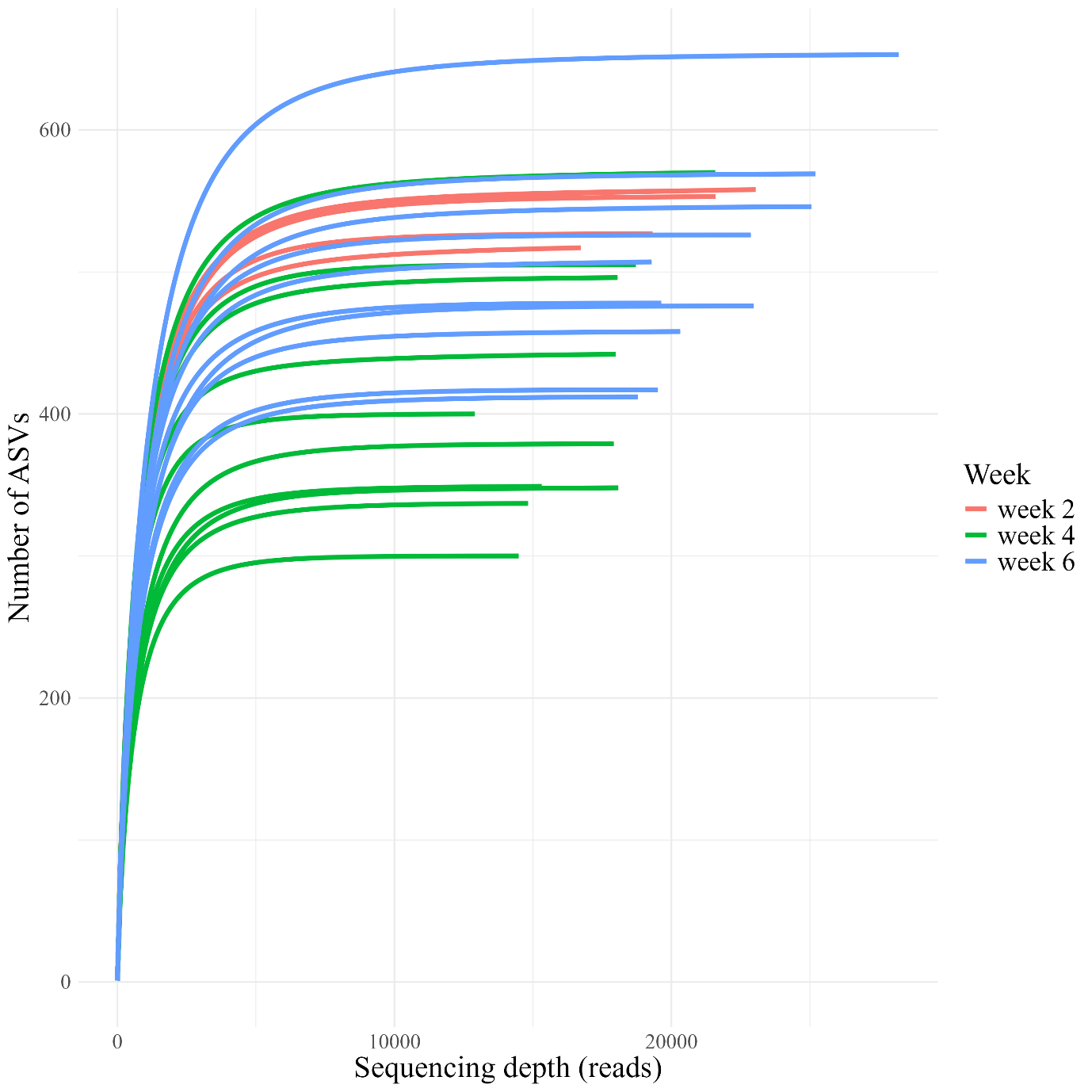


Figure S3. Rarefaction curves of the 16S rRNA samples.


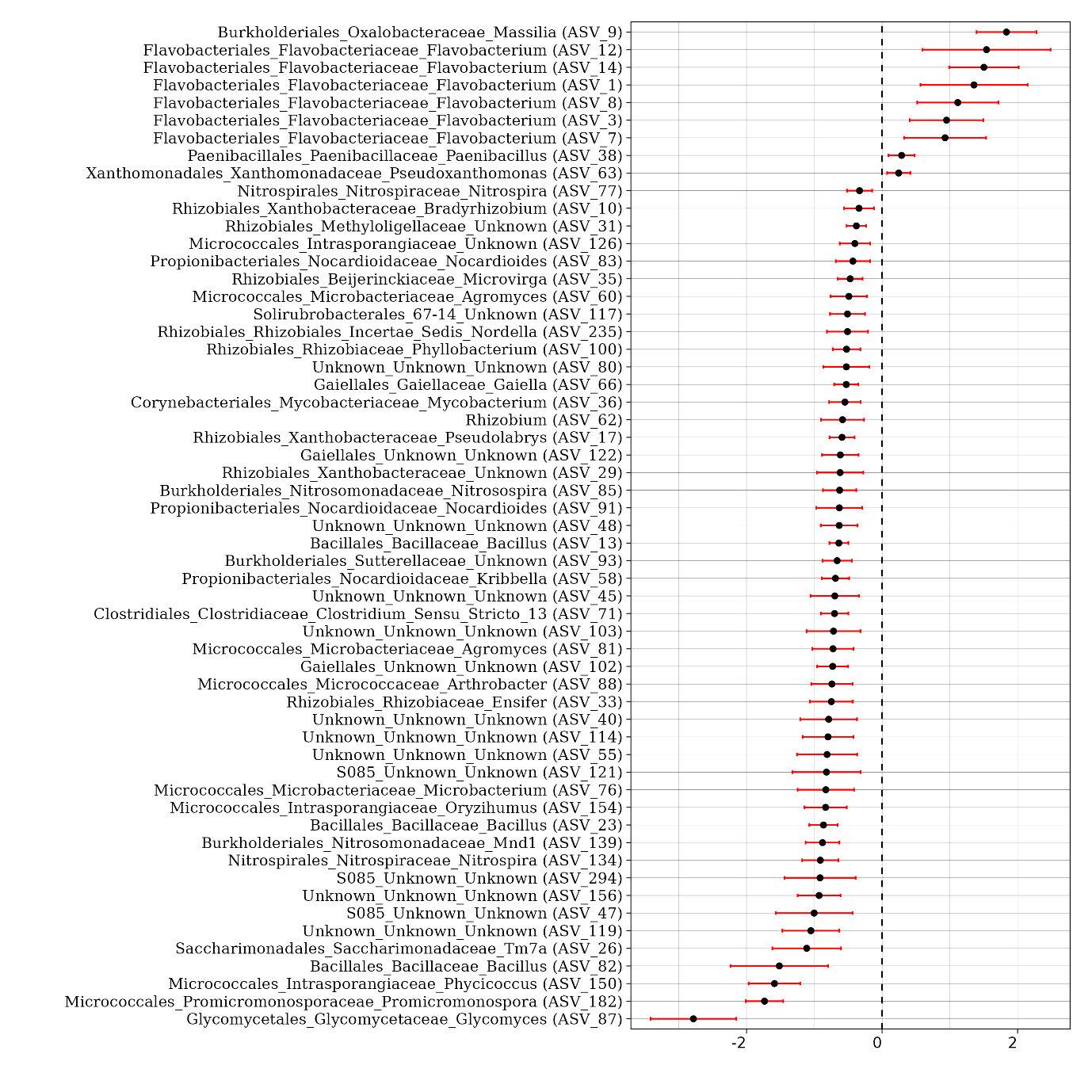


Figure S4. Differentially abundant bacterial ASVs between drought-stressed plants and control plants at week 4 determined by corncob, FDR < 0.05. Symbols on the right of the dashed line indicate an increase in relative abundance in the rhizoplane of the control plants compared to drought-stressed plants, whereas symbols on the left side indicate a higher relative abundance in drought-stressed plants.


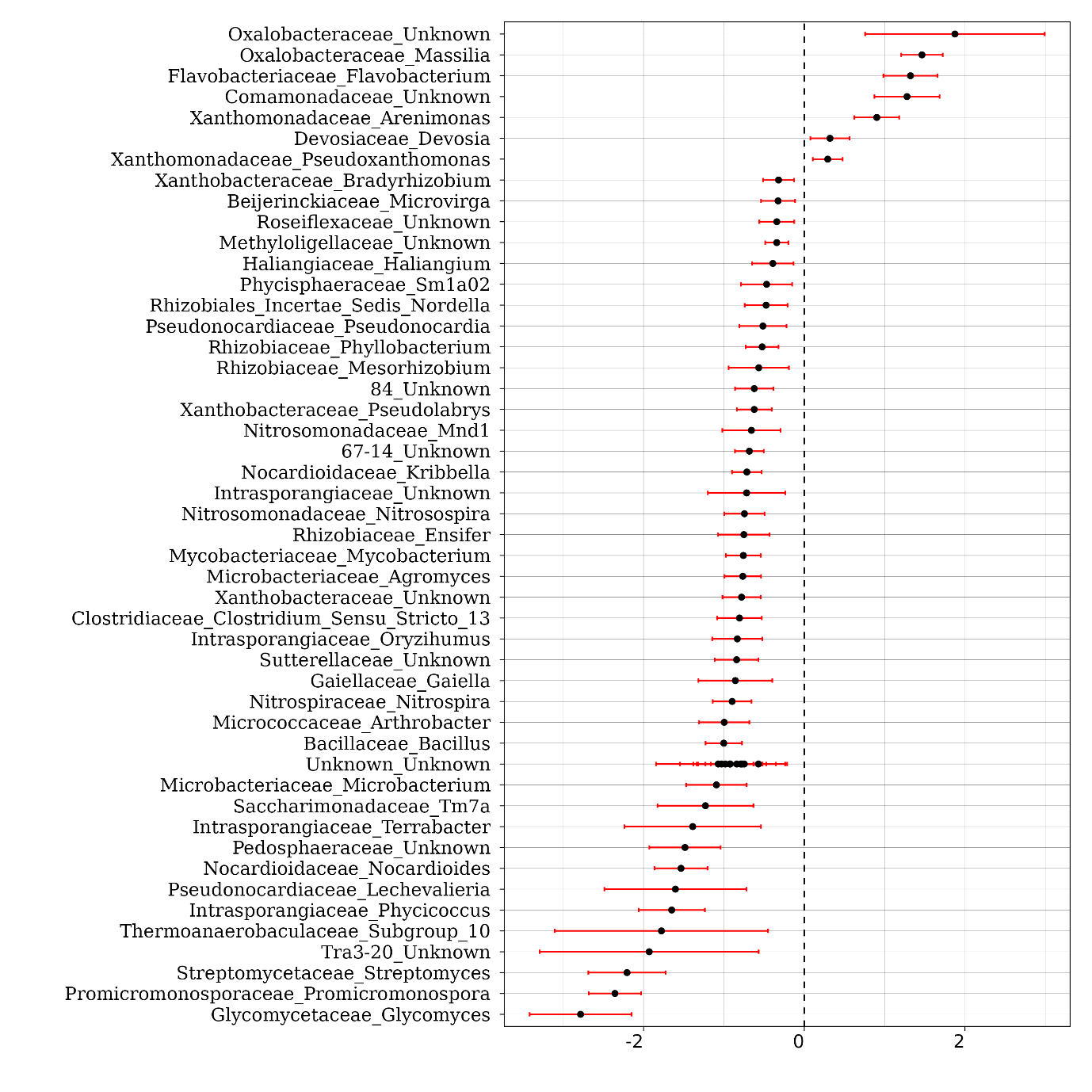


Figure S5. Differentially abundant genera between drought-stressed plants and control plants at week 4 determined by corncob, FDR < 0.05. Symbols on the right of the dashed line indicate an increase in relative abundance in the rhizoplane of the control plants compared to drought-stressed plants, whereas symbols on the left side indicate a higher relative abundance in drought-stressed plants.


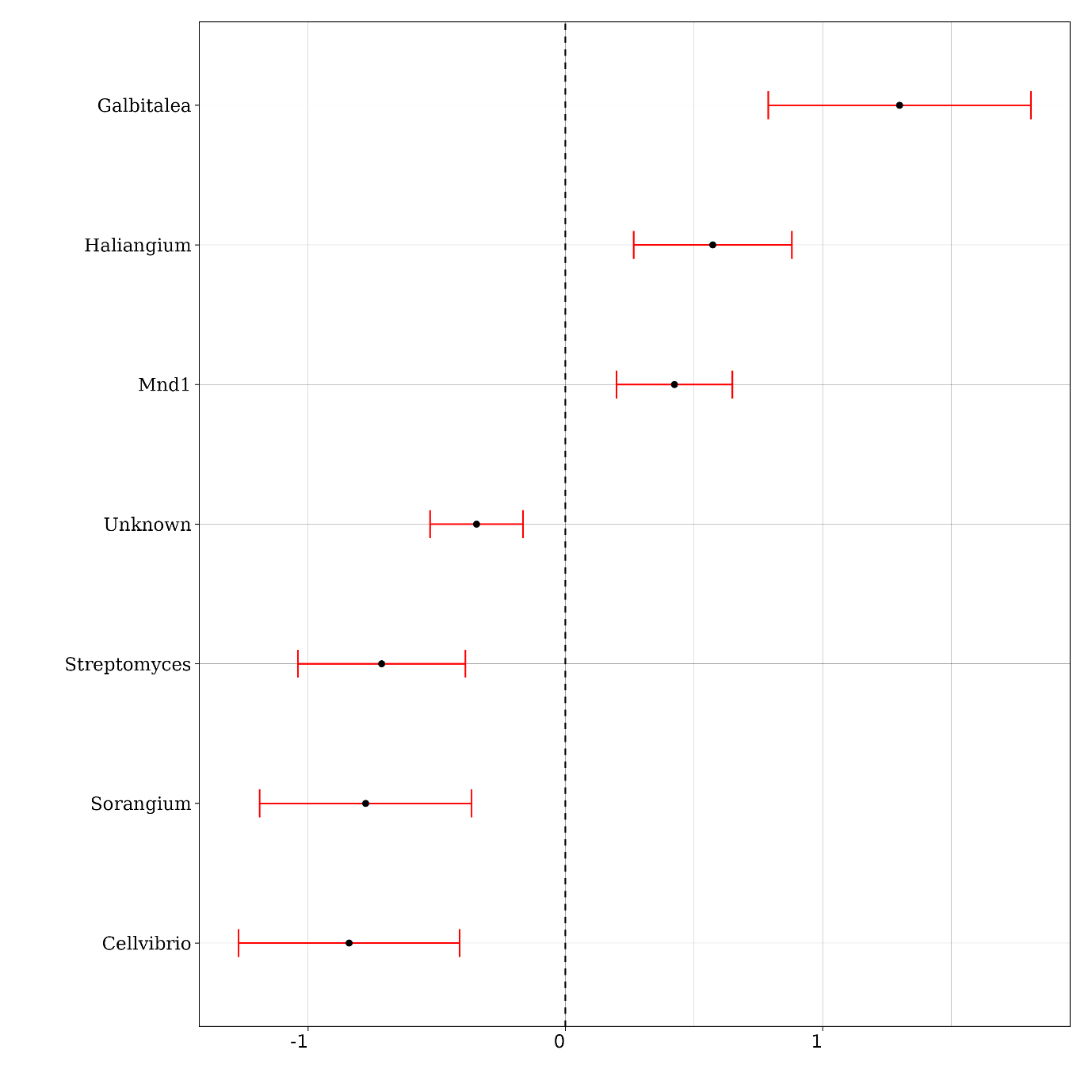


Figure S6. Differentially abundant genera between drought-stressed plants and control plants at week 6 determined by corncob, FDR < 0.05. Symbols on the right of the dashed line indicate an increase in relative abundance in the rhizoplane of the control plants compared to drought-stressed plants, whereas symbols on the left side indicate a higher relative abundance in drought-stressed plants.

| 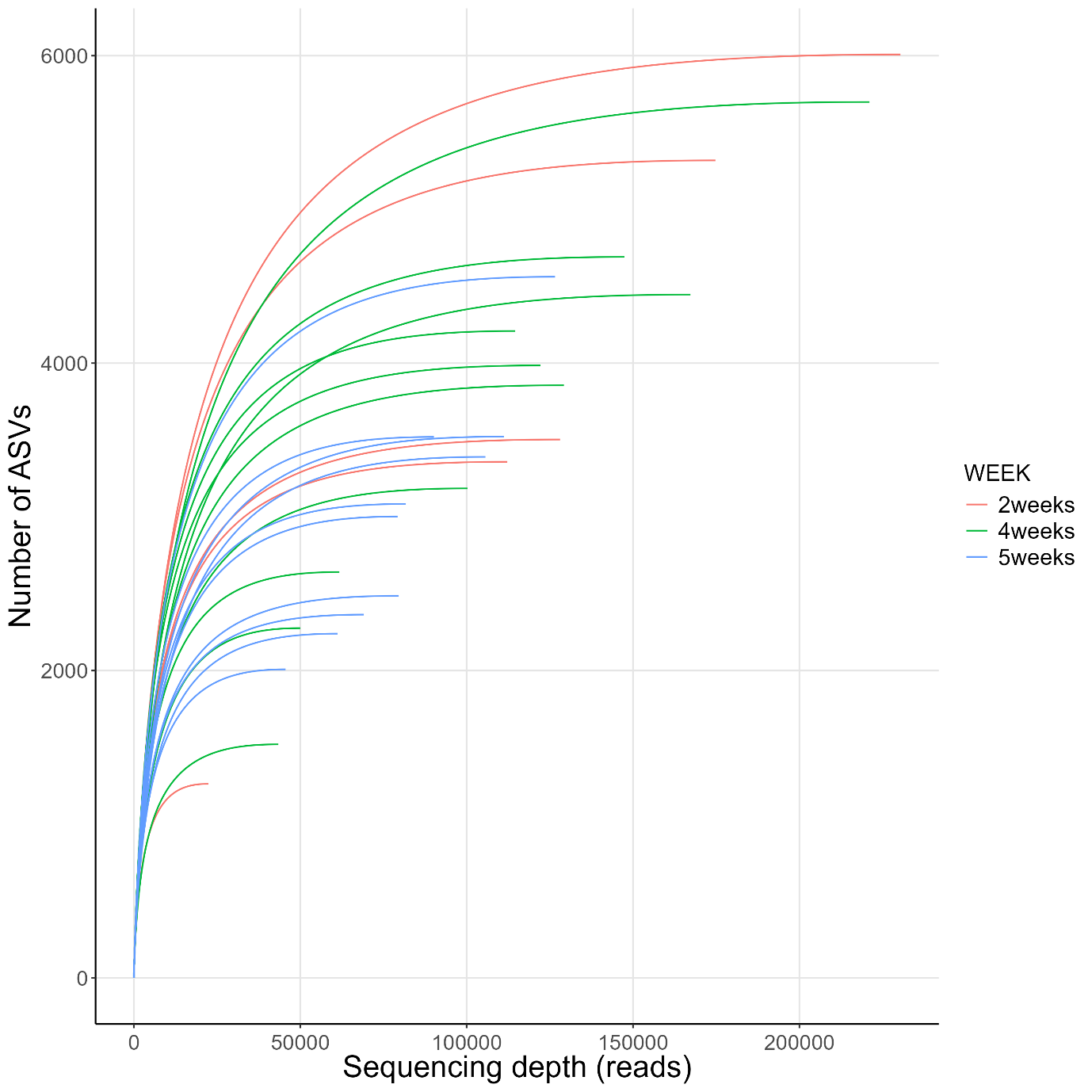 |  |
| --- | --- |

Figure S7. Rarefaction curves of the NRPS samples rhizoplane.


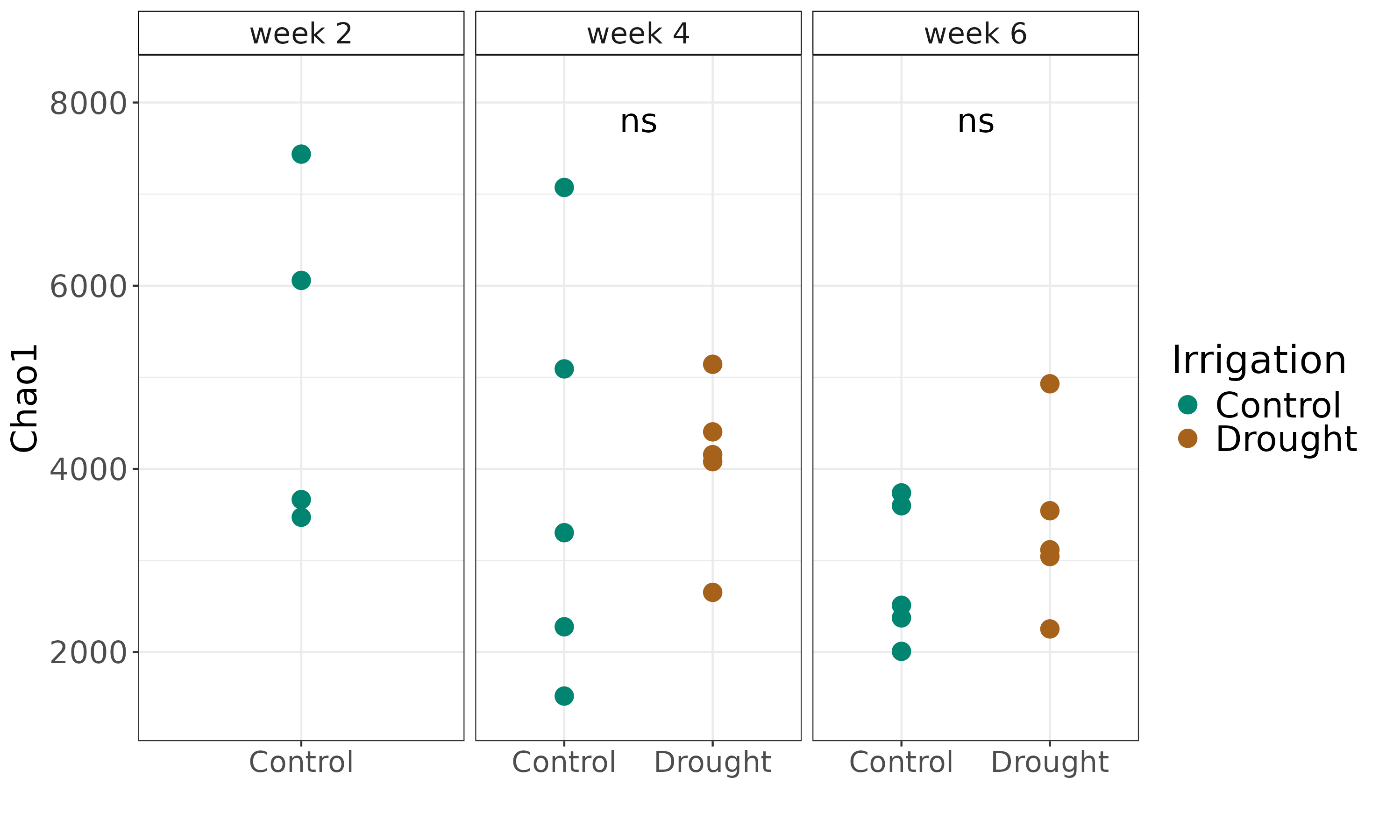


Figure S8. Chao1 richness of the NRPS ACs in the rhizoplane samples from control and drought stressed plants. NS = not significant (p > 0.05, t-test).


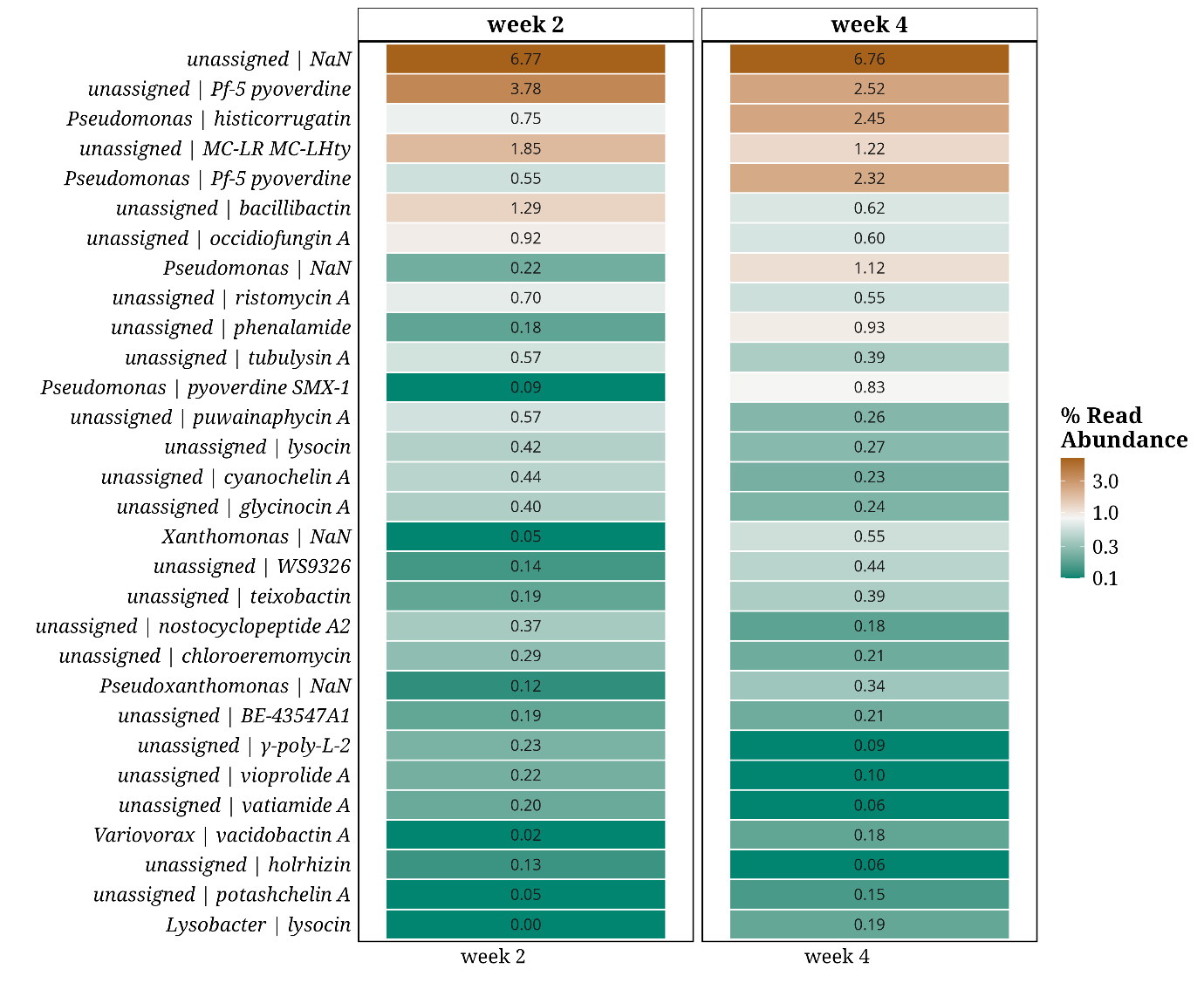


Figure S9. The 30 most abundant NRPS ACs which were differentially abundant between week 2 and 4 under control conditions. Differentially abundance was determined by corncob, FDR < 0.05. Values are mean relative abundances (n = 5).


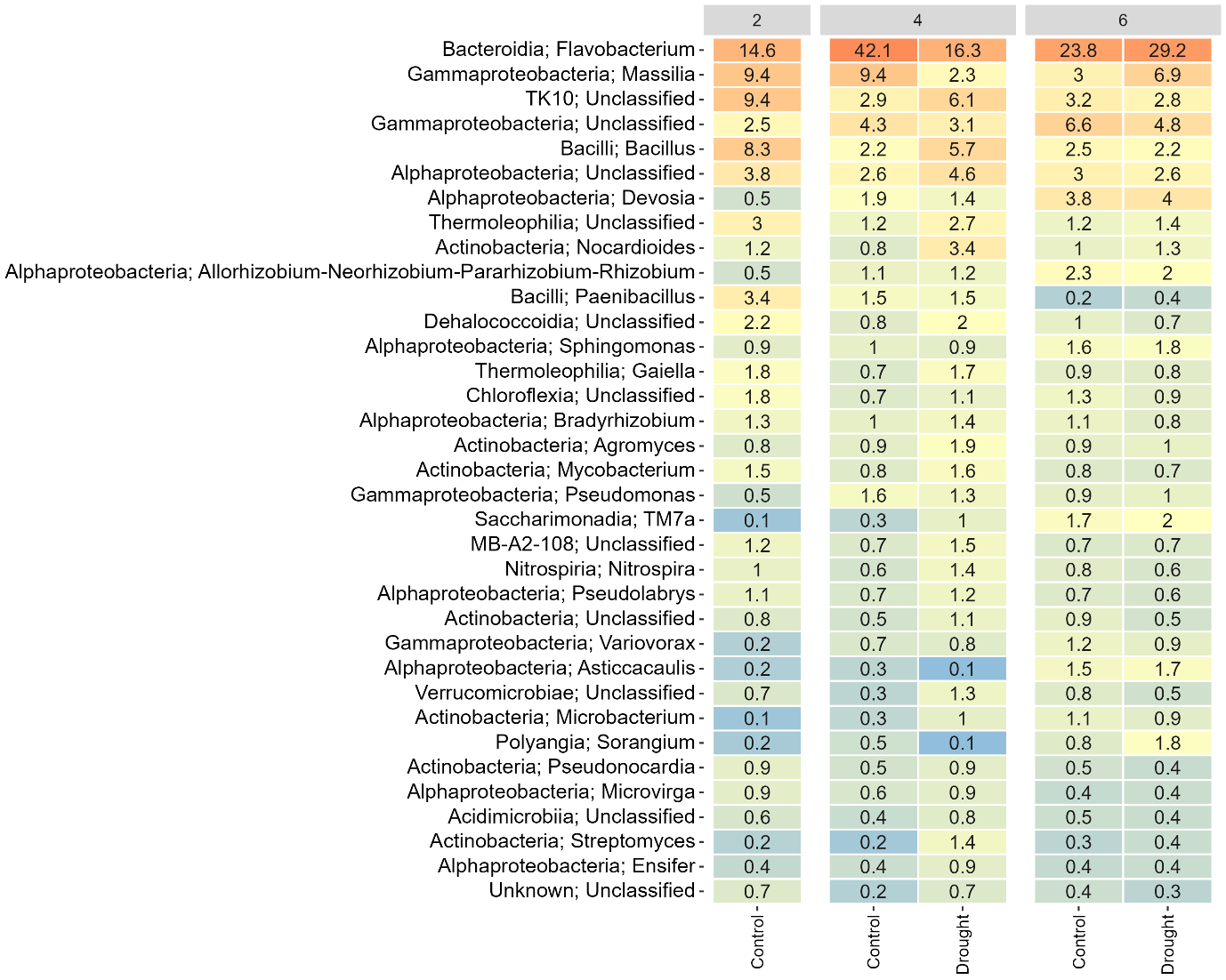


Figure S10. The 35 most abundant genera based on 16S rRNA amplicon sequencing across time and watering condition in the wheat rhizoplane (n = 5).


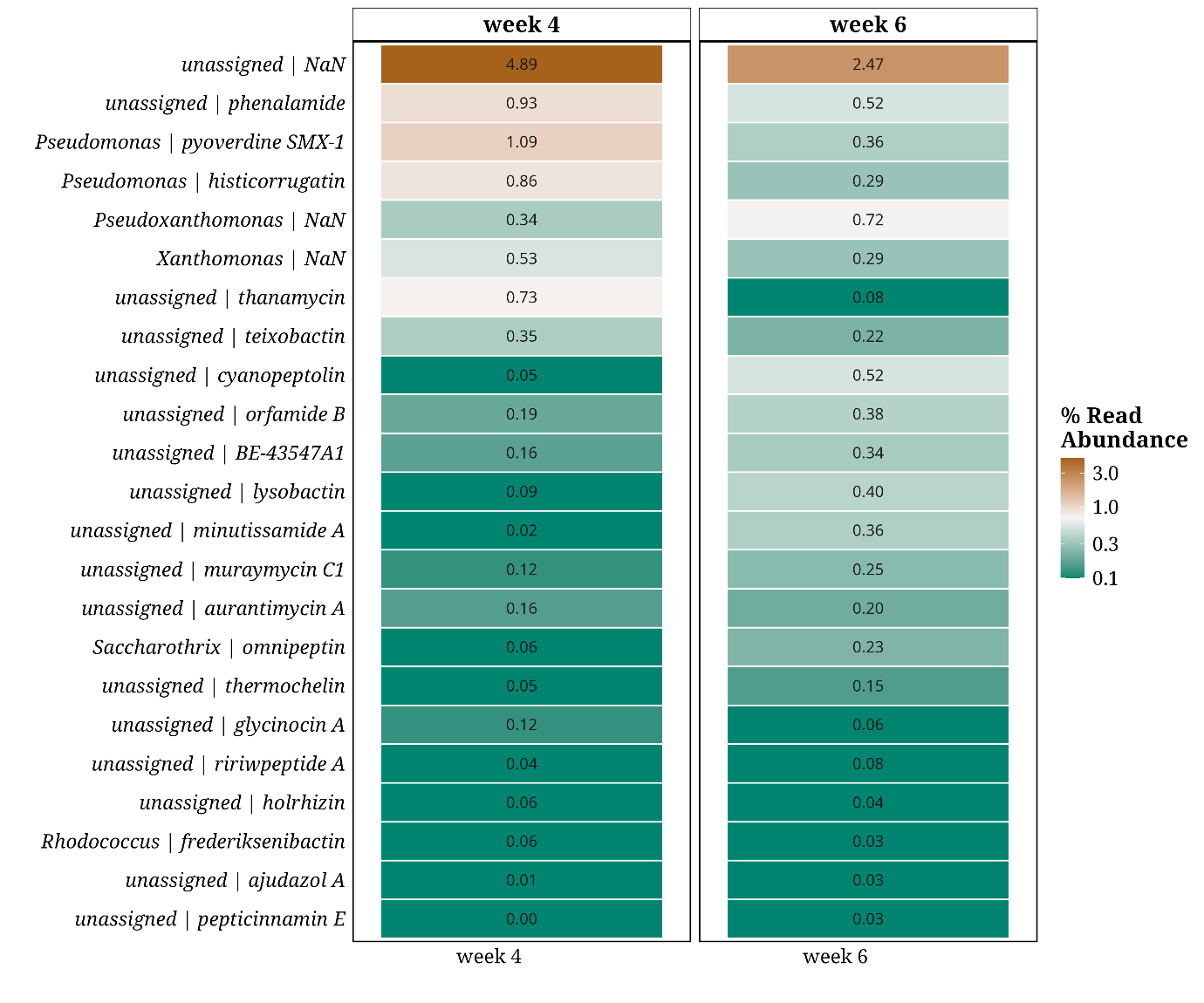


Figure S11. The 30 most abundant NRPS ACs which were differentially abundant between week 4 and 6 under control conditions. Differentially abundance was determined by corncob, FDR < 0.05. Values are mean relative abundances (n = 5).


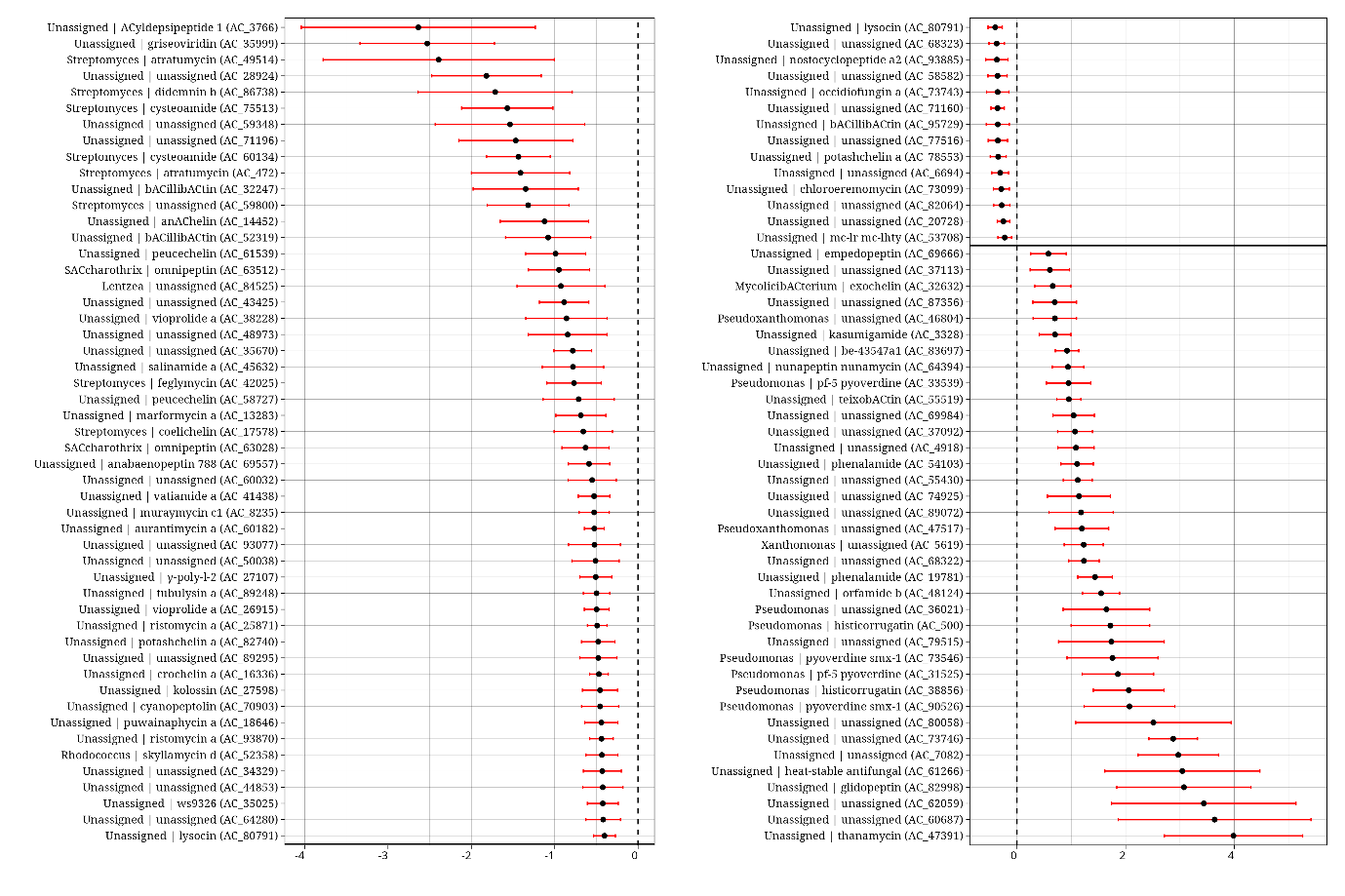


Figure S12. Differential abundant ACs between drought-stressed plants and control plants at week 4 determined by corncob, FDR < 0.05. Symbols on the right of the dashed line indicate an increase in relative abundance in the rhizoplane of the control plants compared to drought-stressed plants, whereas symbols on the left side indicate a higher relative abundance in drought-stressed plants.


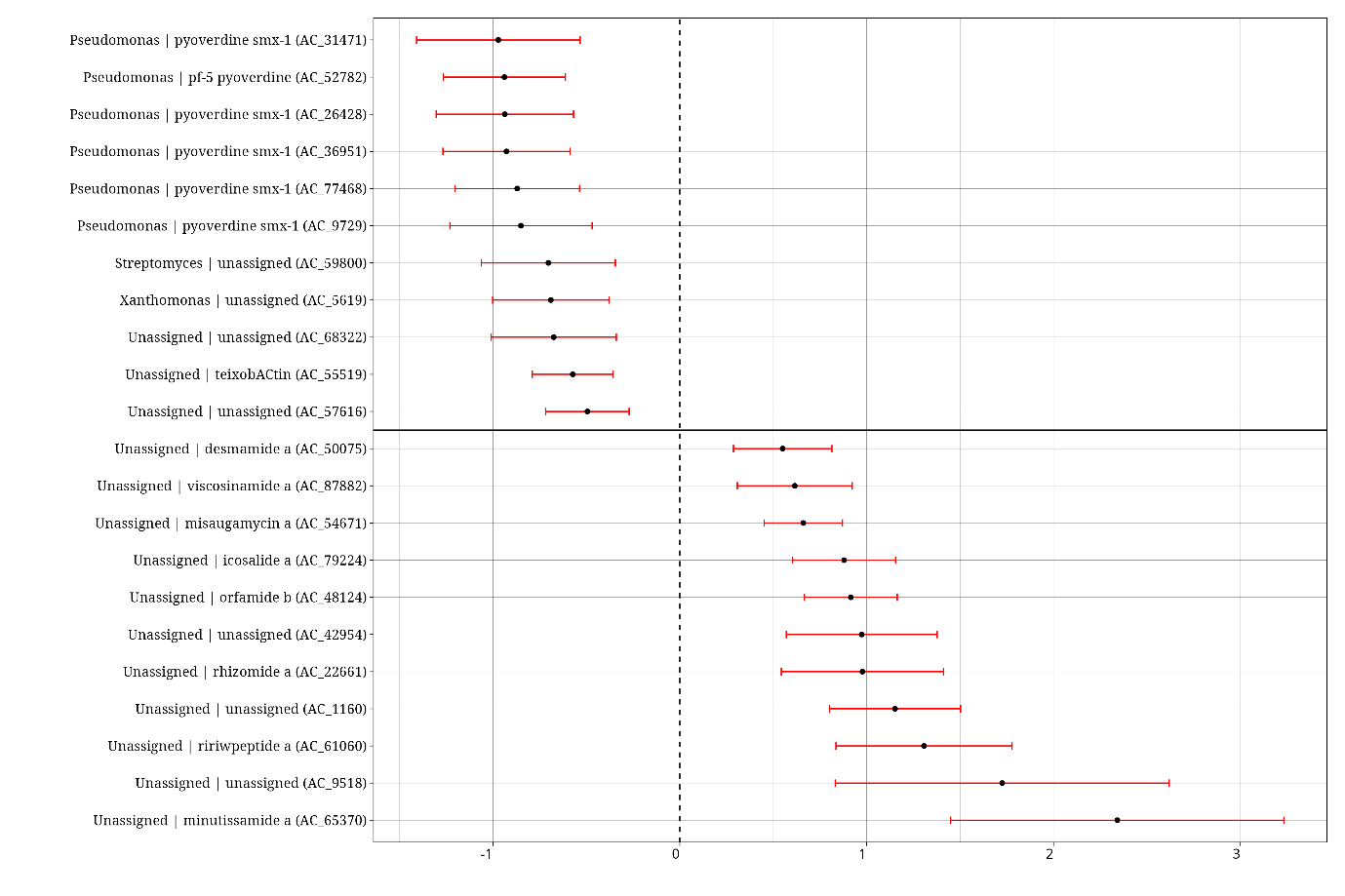


Figure S13. Differential abundant ACs between drought-stressed plants and control plants at week 6 determined by corncob, FDR < 0.05. Symbols on the right of the dashed line indicate an increase in relative abundance in the rhizoplane of the control plants compared to drought-stressed plants, whereas symbols on the left side indicate a higher relative abundance in drought-stressed plants.


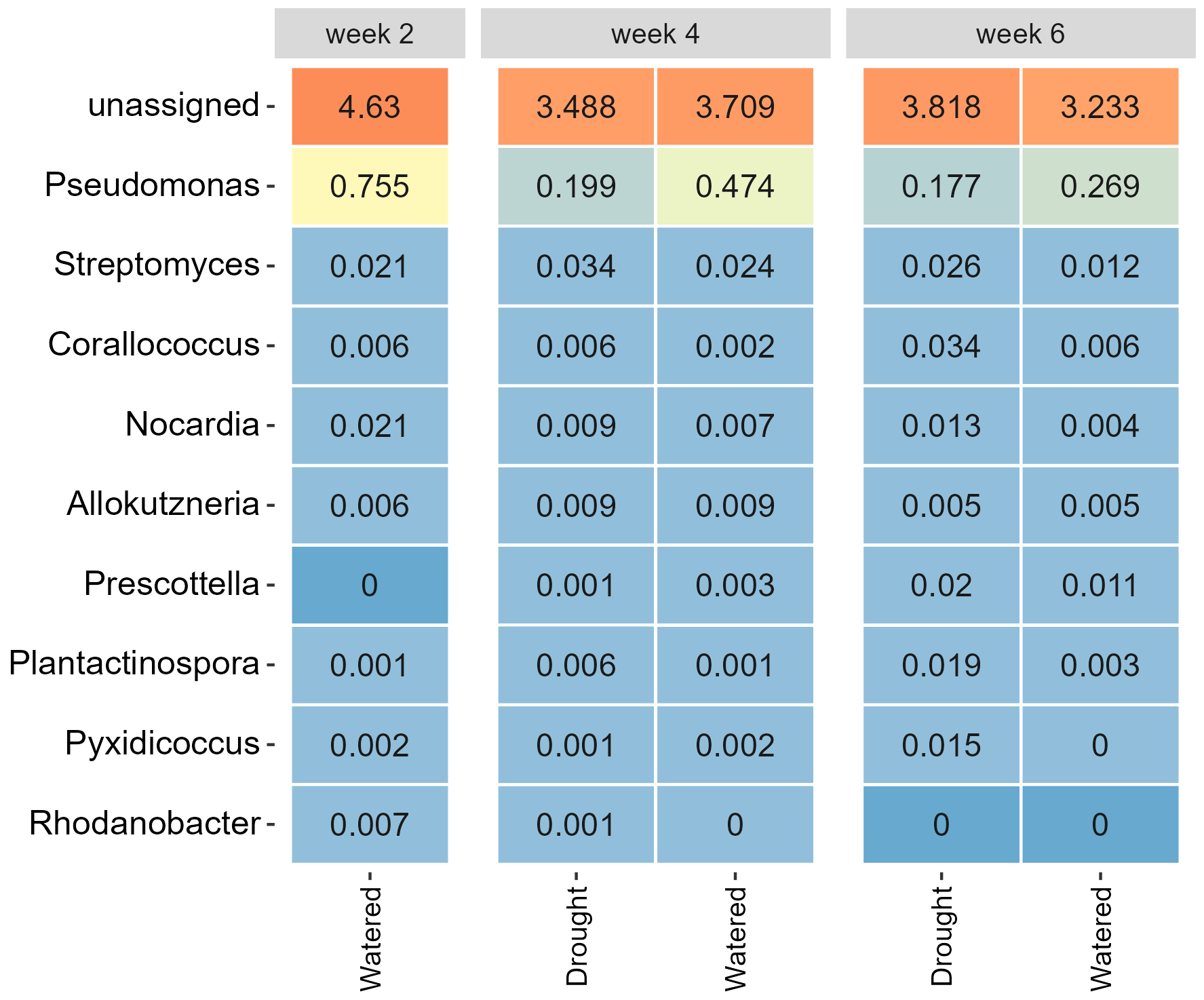


Figure S14. The relative abundances of the genera with the highest proportion of CLPs based on MiBig blast results. The relative abundance is of all NRPS ACs.

Supporting Methods

**Chlorophyll quantification**

The chlorophyll content of leaves of both well-watered and drought-stressed plants was evaluated at 28 dpi using a modified version of Liang's method (Liang *et al.*, 2017). For the assessment, an exact weight of 0.1 g of leaf tissue was obtained, followed by finely chopping the tissue and immersing it in 10 ml of 80% acetone. These samples were then stored in darkness for 48 hours and subsequently centrifuged at a speed of 14,000 g for 5 minutes to achieve a clear mixture. The chlorophyll content was then calculated employing Arnon’s classic equations (Arnon, 1949) with absorbance values measured at 663 nm and 645 nm. The equations used are as follows:

Total chlorophyll (mg/L) = 20.21 (A645) + 8.02 (A663)

Chlorophyll content (mg/g) = C×V/A×1000

Where:

C: chlorophyll concentration in mg/L

V: total volume of extraction solution in ml

A: fresh weight of the sample in g

**Determination of Peroxidase (POD) and Superoxide Dismutase (SOD) activities in wheat leaves**

SOD and POD activities were measured at 28 dpi, following a slightly modified version of the method previously described (Prochazkova *et al.*, 2001). An exact weight of 0.1 g of leaf material was ground into a homogenous suspension with 2 ml of cooled extraction medium (phosphate buffer with 1% polyvinylpyrrolidone, pH 7.8) in a chilled mortar. The extraction medium was used to rinse the mortar, bringing the final volume to 10 ml. After centrifuging the mixture at 4°C, 10000 rpm for 15 minutes, the resulting supernatant was utilized as the enzyme source.

SOD activity was estimated by recording the reduction in the optical density of the nitro-blue tetrazolium (NBT) dye catalyzed by the enzyme. Three milliliters of the reaction mixture, comprising 130 mM methionine, 0.75 mM nitroblue tetrazolium chloride, 0.1 mM EDTA-Na_2_, 50 mM phosphate buffer (pH 7.8), and 0.1 ml enzyme, was initiated by adding 20 μM riboflavine and placing the tubes under fluorescent lamps for 25 minutes. The reaction was halted by switching off the light and storing the tubes in darkness. A complete reaction mixture without the enzyme, which yielded the maximum color, was used as a control, and a non-irradiated complete reaction mixture served as a blank. The optical density was documented at 560 nm, and a single unit of enzyme activity was considered as the quantity of enzyme that diminished the absorbance value by half relative to test tubes devoid of the enzyme.

POD activity was assessed based on the increase in optical density resulting from the formation of tetra-guaiacol. The 2.5 ml reaction mixture contained 3.4 mM guaiacol, 18 mM H_2_O_2_, 0.2 M phosphate buffer (pH 6.0), and 25 μl enzyme. The optical density resulting from the generation of tetra-guaiacol was measured at 470 nm, and a single enzymatic unit (u) was characterized as a 0.01 increment in the absorbance measurement per minute.
